# Climate-Aware Self-Retrospective Representation Learning for Spatio-Temporal Epidemic Forecasting †

**DOI:** 10.3390/tropicalmed11090240

**Published:** 2026-08-24

**Authors:** Qi Yuan, Han Shu, Yizhi Pan, Tianshuo Li, Hangyi Shen, Weiqi Jiang, Zidan Zhu, Pengpeng Zhang, Ningli Xi, Junyi Xin, Kai Li, Guanqun Sun

**Affiliations:** 1 School of Information Engineering, Hangzhou Medical College, Hangzhou 311399, China; yuanqi@hmc.edu.cn (Q.Y.); litianshuo@hmc.edu.cn (T.L.); shenhangyi@hmc.edu.cn (H.S.); jiangweiqi@hmc.edu.cn (W.J.); zhuzidan@hmc.edu.cn (Z.Z.); zhangpengpeng@hmc.edu.cn (P.Z.); ningli.xi@hmc.edu.cn (N.X.); 2 School of Information Science, Japan Advanced Institute of Science and Technology, Nomi 9231292, Ishikawa, Japan; shu.han@jaist.ac.jp (H.S.); panyizhi@jaist.ac.jp (Y.P.)

**Keywords:** epidemic forecasting, spatio-temporal graph neural networks, meteorological-view predictive graph, retrospective temporal modeling

## Abstract

Spatio-temporal epidemic forecasting aims to predict future outbreak trajectories across interconnected regions from historical epidemiological observations and meteorological covariates. However, existing approaches often fail to preserve historically salient epidemic states or to fully exploit delayed and region-varying meteorological associations, leading to unstable temporal representations and insufficient meteorological-context-aware spatio-temporal context for prediction at later forecast horizons. In this paper, we propose CASRL, a **C**limate-**A**ware **S**elf-**R**etrospective Representation **L**earning network for stable and meteorological-context-aware spatio-temporal epidemic forecasting. CASRL first employs a Self-Retrospective Epidemic Encoder (SREE) to retrospectively aggregate historically salient epidemic states through query-guided weighting and adaptive gating, thereby preserving informative historical epidemic states within the look-back window. It then introduces a Climate-Adaptive Graph Message Passing (CAGMP) module that breaks away from traditional passive feature concatenation. Instead, it constructs a separate meteorological-view predictive graph conditioned on the static spatial prior and adaptively fuses it with the incidence-associated topology to model complex cross-regional predictive associations. By integrating self-retrospective epidemic representations with meteorological-view spatio-temporal interactions, CASRL produces forecasts with improved predictive stability at later forecast horizons. Extensive experiments on two public influenza benchmarks show that CASRL is competitive at shorter forecast horizons and provides clearer advantages at later forecast horizons, particularly in phase-alignment-related evaluation and 15-week-ahead forecasting.

## 1. Introduction

Spatio-temporal epidemic forecasting aims to predict future outbreak trajectories across interconnected regions from historical epidemiological observations and exogenous environmental signals [1,2]. As an important application of AI-driven public health modeling, this task lies at the intersection of epidemic intelligence, spatio-temporal learning, and climate-sensitive disease analytics [3,4]. Reliable forecasts across multiple weekly horizons can support earlier situational awareness, improve the allocation of vaccines and medical resources, and provide public health authorities with actionable lead time for outbreak preparedness. However, this task remains highly challenging, as a reliable model must not only preserve the decaying intrinsic momentum of epidemic dynamics as the forecasting horizon increases, but also leverage lagged meteorological context and dynamic cross-regional predictive associations to improve epidemic forecasting across multiple weekly horizons.

There are various efforts to advance spatio-temporal epidemic forecasting. Early deep learning approaches primarily utilized recurrent neural networks and temporal convolutional architectures to capture longitudinal dependencies in epidemiological series [5,6]. To account for spatial spreading across interconnected regions, Spatio-Temporal Graph Neural Networks (STGNNs) have subsequently become the dominant paradigm by integrating graph message passing with temporal encoders [4,7]. Recent innovations have further refined these architectures by exploring multi-scale transmission patterns and regional heterogeneity through adaptive graph learning mechanisms [8,9].

Another line of work focuses on leveraging climate data to improve epidemic forecasting. Previous epidemiological studies have reported associations between meteorological conditions, particularly absolute humidity and temperature, and influenza transmission intensity and seasonal timing [2,10]. Building on these findings, modern forecasting frameworks have increasingly integrated high-resolution environmental indicators as exogenous covariates for respiratory infection forecasting [1,11]. These studies suggest that meteorological covariates can provide useful auxiliary predictive context for epidemiological forecasting, while mechanistic interpretation requires external validation [12,13,14].

**Figure 1:**
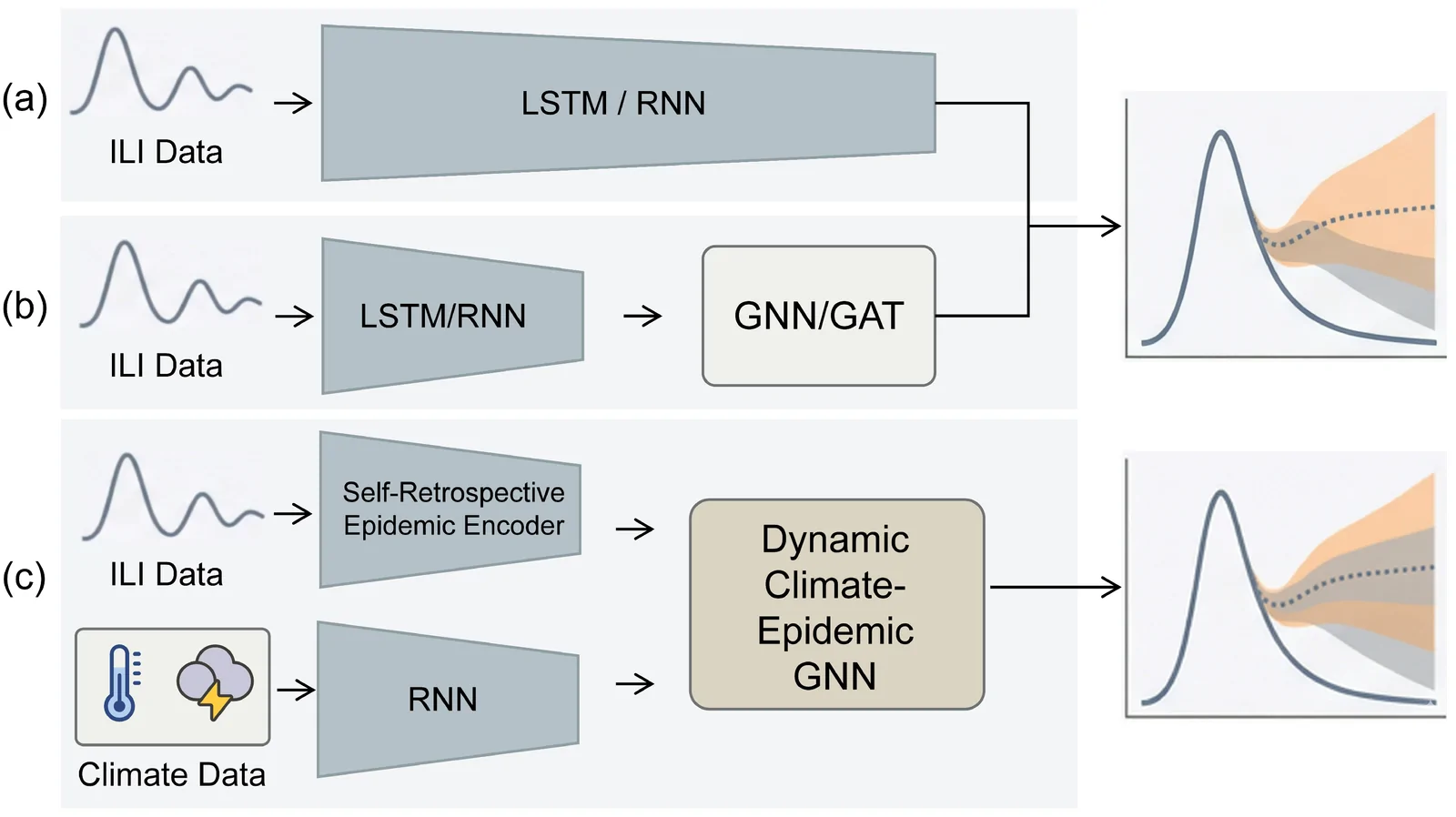
Structural comparison of ILI forecasting paradigms. (**a**) LSTM/RNN: failing to preserve distant historical states. (**b**) Standard STGNN: environmentally-blind with decaying stability. (**c**) CASRL (Ours): achieving stable prediction via self-retrospective learning and climate-aware exogenous meteorological signals.

Despite these advances in spatio-temporal epidemic forecasting, existing methods still suffer from two limitations. First, although current epidemic forecasting methods can capture temporal patterns from historical influenza series, their temporal representations often fail to preserve historically salient epidemic states. This is because commonly used sequence encoders, such as RNNs, LSTMs, or generic Transformers [15,16,17,18], are not explicitly designed to retain earlier but predictively informative states within the historical input window, as shown in Figure 1a,b. As a result, these models may overemphasize recent observations while underutilizing earlier but predictively informative outbreak signals.

Second, existing methods that incorporate climate information typically focus on short-term forecasting or geographically limited scenarios, rather than spatio-temporal forecasting across multiple weekly horizons and interconnected regions. Consequently, they may fail to capture delayed weather effects at later forecast horizons and underexploit regional meteorological heterogeneity associated with influenza dynamics. In spatio-temporal epidemic forecasting, meteorological information should be modeled not merely as passive numerical covariates, but as a structured auxiliary predictive context that captures climate-associated similarities among regions, as exemplified in Figure 1c.

These limitations motivate two design considerations. First, reliable spatio-temporal epidemic forecasting requires more than generic sequence modeling; it demands the preservation of historically salient epidemic states that remain predictively informative far beyond the most recent observations. Moreover, epidemic evolution is inherently retrospective: future outbreak trajectories are often shaped by the interaction between recent transmission momentum and earlier historical states that encode prior transition patterns. Therefore, it is important to explicitly retrieve and preserve informative historical states within the input window to improve the stability of temporal representations at later forecast horizons.

Second, meteorological information should not be viewed merely as a peripheral auxiliary feature, but as a structured predictive context that may complement historical incidence patterns. Because meteorological similarity may be associated with temporally aligned epidemic patterns across regions, climate data can provide an auxiliary spatial context for predictive modeling. By employing a node-specific gating mechanism to adaptively fuse incidence-associated and meteorological-view graph representations, the forecasting architecture can capture predictive spatial associations that are not fully represented by a fixed geographic graph.

**Figure 2:**
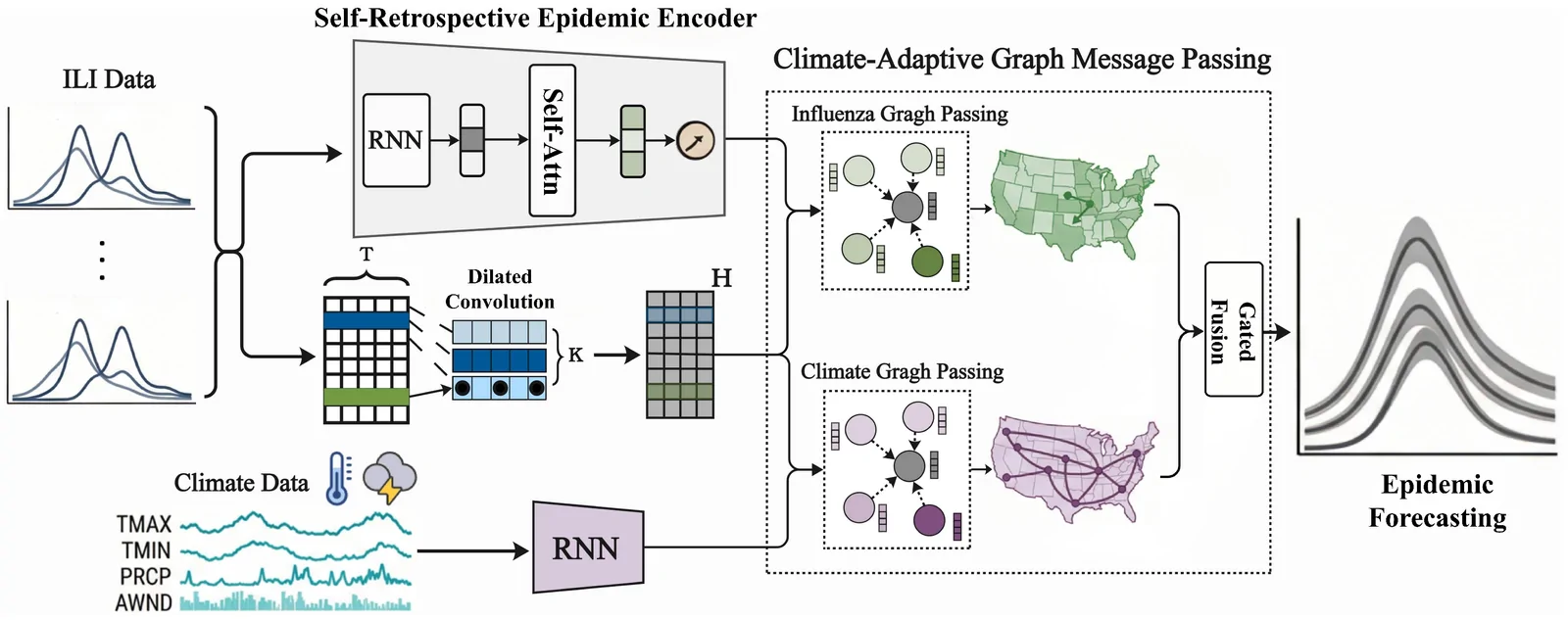
Overall architecture of the proposed CASRL framework. The SREE module extracts historically salient epidemic states via query-guided weighting. Simultaneously, meteorological representations and localized incidence features are encoded through a dedicated RNN and dilated convolutions, respectively. The CAGMP module then executes adaptive spatial message passing across the two predictive graph views, yielding aggregated representations for spatio-temporal epidemic forecasting.

Driven by these insights, we propose a novel **C**limate-**A**ware **S**elf-**R**etrospective Representation **L**earning network (CASRL) for spatio-temporal epidemic forecasting, as illustrated in Figure 2. Unlike standard STGNNs that rely on a single mixed dynamic graph, CASRL introduces a Dual-Topology Decoupling mechanism to prevent the dominant historical incidence from masking weak environmental signals. Specifically, to mitigate autoregressive decay, we first design a Self-Retrospective Epidemic Encoder (SREE) to extract and preserve historically salient epidemic states. Next, a Climate Feature Encoder captures climatic accumulation through a standard RNN, which informs an Adaptive Dual-Graph Construction module to generate incidence-associated and meteorological-view predictive topologies. Finally, the Climate-Adaptive Graph Message Passing (CAGMP) module leverages these dual-topological views to mediate regional interactions between incidence-associated and meteorological-view message passing. Through this unified architecture, CASRL learns discriminative spatio-temporal representations for forecasting across interconnected regions.

The main contributions of this work can be summarized as follows.

1. We propose CASRL, a Climate-aware Self-Retrospective Representation Learning framework designed to address autoregressive decay and spatial rigidity in spatio-temporal epidemic forecasting through the joint modeling of historical momentum and climatic signals.
2. We devise a decoupled dual-view representation paradigm that leverages SREE for retrospective feature extraction while encoding meteorological covariates through a separate climate pathway for adaptive dual-graph construction. Through the CAGMP module, the framework adaptively integrates incidence-associated and meteorological-view predictive topologies to produce discriminative spatio-temporal representations.
3. Extensive experiments on two public ILI forecasting benchmarks show that CASRL achieves competitive performance at shorter forecast horizons, and clearer advantages at later forecast horizons, particularly for PCC-based phase alignment and 15-week-ahead forecasting. The results indicate the value of incorporating meteorological-view predictive context for spatio-temporal epidemic forecasting across multiple weekly horizons.

## 2. Related Work

### 2.1. Spatio-Temporal Deep Learning for Epidemic Forecasting

Deep learning paradigms, particularly Spatio-Temporal Graph Neural Networks (STGNNs), have emerged as powerful tools for epidemic forecasting by capturing non-linear temporal dynamics and cross-regional spatial dependencies [4,7,8,9,19]. Pioneering architectures established the foundation for this data-driven approach. Notably, ColaGNN [7] introduced a cross-location attention mechanism to learn dynamic graph structures for long-term ILI prediction, while STAN [4] integrated epidemiological constraints into graph attention networks to explicitly model spatial transmission risks. Building upon these seminal works, recent advancements have increasingly focused on capturing complex topological heterogeneity. For instance, EpiGNN [8] was proposed to assess regional transmission risks via region-aware graph learners, and MSGNN [9] was developed to exploit multi-scale epidemic patterns through temporal CNNs and stacked attention-based GNN layers. Furthermore, HeatGNN [19] computes time-varying mechanistic affinity graphs to encode spatial heterogeneity. Beyond graph-based spatial modeling, temporal representation learning is another important direction for epidemic forecasting. Existing recurrent and attention-based models summarize historical incidence trajectories, but their representations can still be dominated by recent observations when the forecasting lead time increases. Our previous RAM module explored query-guided retrospective weighting to retrieve informative historical epidemic states for forecasting [20]. Building on this temporal representation perspective, SREE organizes the retrieved historical epidemic states into node-wise epidemic representations, which are then used for incidence-associated graph construction and further integrated with the meteorological-view pathway.

Despite these sophisticated architectural evolutions, current spatio-temporal forecasting models still share an important methodological limitation: they primarily rely on internal spatial interactions and historical autoregression. This makes their use of exogenous meteorological context limited. When evaluated at later forecast horizons (e.g., up to 15 weeks), the historical infectious momentum inherently decays. Without auxiliary meteorological covariates, purely autoregressive STGNNs and incidence-only retrospective models may become less stable at later forecast horizons, especially when near-term incidence signals become insufficient.

### 2.2. Climate-Aware Influenza-like Illness Prediction

To improve epidemic forecasting with external context, an emerging body of literature has increasingly incorporated climate variables as auxiliary meteorological covariates to predict climate-sensitive outbreaks. While these studies suggest the potential value of meteorological covariates, they exhibit three methodological limitations when confronted with spatio-temporal predictive modeling. First, they are heavily restricted by short-term temporal horizons. For instance, Koge et al. [21] and Lueangwitchajaroen et al. [22] recently integrated climate data with LSTM and transfer learning architectures, respectively. However, their evaluations are strictly confined to 1-to-4 week near-term predictions. Within such brief windows, historical infectious momentum dominates the prediction, masking the severe “autoregressive decay” that inevitably degrades model performance over extended periods. Second, existing frameworks fundamentally lack complex spatial topology. Recent applications by Koge et al. [21], Amendolara et al. [23], and Tuan et al. [24] utilized deep learning for ILI and multi-disease forecasting; yet, their scopes were narrowly restricted to isolated single urban centers. Even when broader studies expand their geographical scope (e.g., Stamper et al. [25]), they evaluate climate extremes across separate tropical zones in isolation, without modeling cross-regional predictive associations between them. Consequently, these models rely on geographically naive climate averages, ignoring the multi-state spatial heterogeneity associated with regional meteorological conditions. Most critically, current approaches suffer from architectural rigidity regarding feature fusion. The aforementioned models [21,23,24] uniformly treat multi-dimensional climate data merely as passive, concatenated numerical features appended to historical case counts. Relying on static geographical assumptions, they provide limited capacity to represent how meteorological similarity may contribute to spatially heterogeneous predictive patterns.

To bridge this gap, CASRL models meteorological covariates as a separate predictive graph view rather than as only passively concatenated numerical features. By combining SREE with CAGMP, CASRL preserves historical epidemic representations while adaptively integrating incidence-associated and meteorological-view predictive topologies for epidemic forecasting.

## 3. Methodology

We propose the Climate-Aware Self-Retrospective Representation Learning (CASRL) framework to address the inherent autoregressive decay and spatial rigidity in spatio-temporal epidemic forecasting. As illustrated in Figure 2, given the historical incidence $X_{ili}$ and concurrent climate signals $X_{cli}$, the architecture operates through four sequential stages: First, the Self-Retrospective Epidemic Encoder (SREE) utilizes a query-guided weighting mechanism to extract historically informative epidemic states. Second, a Climate Feature Encoder stream processes exogenous signals through a standard RNN to capture climatic accumulation. Third, an Adaptive Dual-Graph Construction module translates these temporal and meteorological representations into incidence-associated and meteorological-view predictive topologies. Finally, the Climate-Adaptive Graph Message Passing (CAGMP) module performs adaptive spatial message passing across these two graph views, which is integrated with retrospective temporal representations to generate the h-week-ahead prediction $Y\in R^{M}$.

### 3.1. Self-Retrospective Epidemic Encoder

State-of-the-art sequence models typically create an information bottleneck by compressing historical time-series into a fixed-size representation or using only the final hidden state of a recurrent neural network. To address this limitation and prevent the loss of crucial sequential dynamics, we propose the SREE. In our framework, “self-retrospective” explicitly refers to the mechanism where the sequence exclusively utilizes its own terminal hidden state as an attention query to adaptively retrieve and weight historically salient states, without relying on external cross-attention. The SREE module uses the terminal hidden state as a query to retrieve historically informative epidemic states for subsequent spatial graph construction. The raw incidence sequence $X_{ili}\in R^{W\times M}$ is first processed by an RNN to generate a full chronological trajectory of hidden states $E=\left[e_{1},\ldots ,e_{W}\right]\in R^{W\times d}$.

Specifically, it uses the final hidden state $e_{W}$ as a dedicated query to assign adaptive weights to all previous states $E_{1:W-1}$ (which serve as keys and values). The attention scores and the resulting context vector $C_{epi}$ are calculated as follows:

(1) $$C_{epi}=\mathrm{Softmax}\left(\frac{e_{W}E_{1:W-1}^{⊤}}{\sqrt{d}}\right)E_{1:W-1}$$

Subsequently, a gated fusion mechanism adaptively combines the original terminal state $e_{W}$ with the context vector $C_{epi}$ to produce the enhanced temporal representation:

(2) $$G_{epi}=\mathrm{Softmax}\left(\mathrm{MLP}\left(\left[C_{epi},e_{W}\right]\right)\right)$$

For notational simplicity, the equations below describe the retrospective weighting process for one spatial node; the resulting node-wise representations are then stacked across all *M* nodes to obtain $H_{epi}\in R^{M\times d}$.

(3) $$H_{epi}=G_{epi}^{\left(1\right)}⊙C_{epi}+G_{epi}^{\left(2\right)}⊙e_{W}$$

This query-guided approach ensures that the fused representation $H_{epi}\in R^{M\times d}$ retains historically informative epidemic representations required for subsequent dynamic graph construction.

### 3.2. Climate Feature Encoder

To incorporate exogenous meteorological covariates while keeping them separated from incidence dynamics before graph construction, we encode the meteorological sequence through a dedicated climate pathway.

The concurrent multivariate climate sequence is denoted as $X_{cli}\in R^{W\times M\times 4}$, where the final dimension comprises four core region-specific environmental indicators: Maximum Temperature (TMAX), Minimum Temperature (TMIN), Precipitation (PRCP), and Average Wind Speed (AWND).

Unlike general time-series forecasting, incorporating meteorological covariates requires capturing delayed and cumulative environmental patterns within the observable look-back window. Rather than relying on static statistical lag assumptions, we process $X_{cli}$ through a dedicated, standard RNN. This recurrent pathway is used to summarize delayed and cumulative meteorological patterns within the observable historical window. We extract the terminal hidden state from this recurrent pathway as the region-specific meteorological representation:

(4) $$H_{cli}={\mathrm{RNN}}_{cli}\left(X_{cli}\right)\left[-1\right]$$

where $H_{cli}\in R^{M\times d}$. By using a separate recurrent pathway, the architecture preserves a clear distinction between incidence-associated and meteorological representations before graph construction. This separation allows the subsequent graph construction to use meteorological information as a distinct predictive view, reducing premature mixing with the dominant autoregressive incidence representation.

### 3.3. Adaptive Dual-Graph Construction

With the temporally enhanced epidemic representation ($H_{epi}$) and the meteorological representation ($H_{cli}$) established by the parallel encoders, we translate these representations into dynamic predictive topologies. Crucially, rather than concatenating these multi-modal features into a single mixed graph learner, which often causes strong autoregressive signals to drown out weaker exogenous meteorological signals, we employ a Dual-Topology Decoupling strategy. Before constructing the two predictive graph views, we define a generic location-aware graph-construction operator G(F) for a node-feature matrix $F\in R^{M\times d}$. This operator first computes a feature-dependent dynamic adjacency matrix and then anchors it to a shared static geographic prior $A_{prior}$ through a non-linear gate:

(5) $$\tilde{A}=\mathrm{Normalize}\left(\mathrm{ELU}\left(FW_{1}^{⊤}⊕FW_{2}^{⊤}+b_{1}\right)V+b_{v},p=2,dim=1\right)$$

where $W_{1},W_{2}\in R^{d\times d}$ and $V\in R^{d\times 1}$ are learnable weights, and ⊕ denotes broadcasted addition. To ensure structural stability, a non-linear gate fuses this dynamic matrix $\tilde{A}$ with the static geographic prior $A_{prior}$:

(6) $$C=\sigma (\tilde{A}W_{b}+w_{b})$$

(7) $$G\left(F\right)=C⊙A_{prior}+\left(1-C\right)⊙\tilde{A}$$

During forward propagation, this generic graph-construction operator is instantiated for two predictive views with view-specific learnable parameters. First, using the epidemic representation $H_{epi}$ yields the incidence-associated spatial graph $A_{epi}$:

(8) $${\tilde{A}}_{epi}=\mathrm{Normalize}\left(\mathrm{ELU}\left(H_{epi}W_{1,epi}^{⊤}⊕H_{epi}W_{2,epi}^{⊤}+b_{1,epi}\right)V_{epi}+b_{v,epi},p=2,dim=1\right),$$

(9) $$C_{epi}=\sigma \left({\tilde{A}}_{epi}W_{b,epi}+w_{b,epi}\right),$$

(10) $$A_{epi}=C_{epi}⊙A_{prior}+\left(1-C_{epi}\right)⊙{\tilde{A}}_{epi}.$$

Concurrently, using the meteorological representation $H_{cli}$ yields the climate-associated predictive graph $A_{cli}$:

(11) $${\tilde{A}}_{cli}=\mathrm{Normalize}\left(\mathrm{ELU}\left(H_{cli}W_{1,cli}^{⊤}⊕H_{cli}W_{2,cli}^{⊤}+b_{1,cli}\right)V_{cli}+b_{v,cli},p=2,dim=1\right),$$

(12) $$C_{cli}=\sigma \left({\tilde{A}}_{cli}W_{b,cli}+w_{b,cli}\right),$$

(13) $$A_{cli}=C_{cli}⊙A_{prior}+\left(1-C_{cli}\right)⊙{\tilde{A}}_{cli}.$$

The two graph views share the same static geographic prior $A_{prior}$ but use separate learnable parameters for their feature-dependent dynamic components. Accordingly, $A_{cli}$ is treated as a climate-associated predictive topology conditioned on the spatial prior, rather than as an epidemiological contact graph.

Because $H_{\mathrm{cli}}$ is recomputed for each rolling input window, $A_{\mathrm{cli}}$ is reconstructed at every forecast origin. The climate-associated graph is therefore time-varying across forecast origins and fixed only within the CAGMP stack for a given input window.

### 3.4. Climate-Adaptive Graph Message Passing and Forecasting

To initialize the high-frequency node attributes for spatial message passing, the raw incidence $X_{ili}$ is concurrently processed through a temporal convolutional pathway (utilizing parallel standard and dilated convolutions) to extract localized structural variations $X_{node}\in R^{M\times k}$. Let $Z^{\left(l\right)}$ denote the node representations at layer *l* (with the initial input $Z^{\left(0\right)}=X_{node}$). The structural messages are first projected into a shared semantic space via a weight matrix $W_{z}$, yielding the support matrix $S=Z^{\left(l\right)}W_{z}$. These messages are then independently aggregated through both topologies:

(14) $$Out_{epi}=A_{epi}S+b_{epi}$$

(15) $$Out_{cli}=A_{cli}S+b_{cli}$$

A critical challenge in multi-graph fusion is preventing one topology from dominating the learning process, which often leads to gradient starvation for the secondary graph. To adaptively balance the spatial information flow between the incidence-associated branch ($Out_{epi}$) and the climate-associated branch ($Out_{cli}$), CAGMP employs a node-specific adaptive gate modulated by a learnable temperature parameter τ:

(16) $$Logits=\frac{\mathrm{MLP}(\mathrm{LayerNorm}(S))}{\mathrm{Softplus}\left(\tau \right)+\epsilon_{1}}$$

(17) $$Gate=\epsilon_{2}+\left(1-2\epsilon_{2}\right)\cdot \sigma \left(Logits\right)$$

where $\epsilon_{1}=10^{-6}$ ensures numerical stability. Crucially, by explicitly setting $\epsilon_{2}=0.05$, we establish a strict mathematical activation bound of [0.05,0.95]. This bounded-gate design prevents complete gate saturation during backpropagation, ensuring that neither graph-view branch is entirely discarded during optimization. The spatial representation is then updated via element-wise gated fusion:

(18) $$Z^{\left(l+1\right)}=\sigma_{act}\left(\mathrm{Dropout}(Gate⊙Out_{epi}+\left(1-Gate\right)⊙Out_{cli})\right)$$

where $\sigma_{act}$ denotes a non-linear activation function such as ReLU.

**Spatio-Temporal Forecasting Head.** Following the stacked CAGMP module, the final spatial features $Z^{\left(L\right)}$ comprehensively encode the dual-topology propagation. To generate the final incidence prediction $\hat{Y}$, we concatenate this deeply aggregated spatial state with the intrinsic temporal momentum $H_{epi}$. This concatenation explicitly bridges the SREE extraction phase with the ultimate projection, ensuring trajectory stability. Furthermore, an autoregressive residual window $W_{res}$ is incorporated to preserve the absolute scale of the original incidence series:

(19) $${\hat{Y}}^{\left(W+h\right)}=W_{out}[Z^{\left(L\right)}\left|\right|H_{epi}]+b_{out}+W_{res\_proj}\left(X_{ili}^{\left(W-W_{res}+1:W\right)}\right)$$

By combining non-linear spatial message passing, retrospective temporal representations, and a shallow autoregressive residual term, this forecasting head integrates adaptive graph-view information while preserving the magnitude scale of the incidence series.

## 4. Experimental Results and Analysis

### 4.1. Datasets

To support climate-aware spatio-temporal epidemic forecasting, we curated a comprehensive, multi-modal spatio-temporal dataset that aligns macro-scale epidemiological surveillance records with station-level meteorological indicators across U.S. state-level and HHS regional surveillance settings. Crucially, we employ a differentiated normalization strategy tailored to the intrinsic statistical properties of each modality: Min-Max scaling is applied to the zero-bounded ILI counts to preserve spatial magnitude differences, whereas region-specific Z-score standardization is utilized for continuous climate data to capture atmospheric anomalies.

To preserve temporal integrity and prevent data leakage, all normalization statistics and imputation parameters were estimated from the training period only and then applied unchanged to the validation and test periods under the chronological 60%/20%/20% protocol.

#### 4.1.1. Epidemiological Surveillance Data

As summarized in Table 1, to evaluate our framework across diverse spatial scales, we utilize two robust benchmarks of influenza-like illness (ILI) activities sourced directly from the U.S. Centers for Disease Control and Prevention (CDC).

To capture finer-grained spatial dynamics, we first utilize the US-States cohort, which records weekly ILI-positive cases across 49 state-level forecasting nodes over a 360-week period (2010–2017). Additionally, to model broader epidemiological trends, the US-Regions cohort comprises weekly patient visitation data from 10 Health and Human Services (HHS) macro-regions over a 15-year span (785 weeks, 2002–2017). Within our framework, each state-level node or HHS region-level node is treated as a distinct node within a dynamic epidemiological graph.

#### 4.1.2. High-Resolution Climate Data

We extracted daily station-level meteorological records from the NOAA Global Historical Climatology Network Daily (GHCN-Daily) database [26,27]. Following recent climate-aware respiratory-disease forecasting studies that incorporate station-level meteorological covariates into epidemic prediction models [21,28], we first considered seven candidate variables: average temperature (TAVG), maximum temperature (TMAX), minimum temperature (TMIN), precipitation (PRCP), snowfall (SNOW), snow depth (SNWD), and average wind speed (AWND).

For the meteorological inputs, station-level NOAA GHCN-D records were mapped to representative state-level entities and then aggregated consistently for the HHS regional cohort, yielding node-aligned meteorological covariates for the two forecasting benchmarks.

The final covariate set was determined a priori based on epidemiological relevance, long-term cross-state data completeness, and redundancy among the candidate variables. No target-dependent statistical filtering was applied. TMAX and TMIN were retained as temperature-related indicators, PRCP was retained as a precipitation-related environmental proxy, and AWND was retained as an auxiliary weather-context variable. TAVG was not retained because it is largely redundant with TMAX/TMIN in our modeling setting, whereas SNOW and SNWD were excluded because their missingness and spatial relevance were uneven across warm and cold climate zones.

Daily meteorological records were aggregated into weekly covariates to match the CDC epidemiological reporting cycle. To preserve regional climate heterogeneity and avoid future information leakage, climate covariates were standardized using region-specific Z-score normalization with parameters estimated from the training period only and then applied unchanged to the validation and test periods.

#### 4.1.3. Spatio-Temporal Dependency Diagnostics

Serial dependence was assessed using lag-1 autocorrelation and Ljung–Box tests, while spatial dependence was measured using weekly Global Moran’s *I*. Joint spatio-temporal dependence was evaluated using a space–time Moran’s *I* that links geographically adjacent nodes across neighboring weeks. Both cohorts show strong serial dependence and significant joint spatio-temporal dependence, whereas same-week spatial autocorrelation is comparatively weak (Table 2).

The results indicate that the two ILI series are dominated by temporal persistence and joint spatio-temporal organization rather than strong contemporaneous spatial clustering.

### 4.2. Experimental Setup

**Implementation Details.** All models were implemented in PyTorch 2.3.1 and executed on an NVIDIA RTX 3090 GPU to ensure computational reproducibility. The CASRL framework was optimized using the Adam algorithm for a maximum of 1500 epochs with a batch size of 32. We employed a fixed learning rate of $10^{-3}$ and a weight decay of $5\times 10^{-4}$. To prevent over-fitting, an early stopping strategy with a patience of 50 epochs was enforced. For temporal encoding, the retrospective window was set to W=20 weeks to capture sufficient historical momentum. The SREE module utilized two RNN layers, while the temporal convolutional stream employed k=10 filters with dilation rates of 1 and 2 to extract multi-scale volatility patterns. The principal structural hyperparameters were selected according to validation performance. The historical look-back window was evaluated over W∈{10,15,20,25,30}, and the number of CAGMP layers over L∈{1,2,3,4,5}.

**Model Optimization.** Given the noisy nature of epidemiological surveillance data, we optimized the network using the $L_{1}$ loss (Mean Absolute Error) as the objective function: $L=\frac{1}{B\times M}\sum_{b,i}\left|{\hat{Y}}_{b,i}-Y_{b,i}\right|$. Compared to $L_{2}$ loss, the $L_{1}$ formulation is more robust to stochastic outlier events, such as retroactive data reporting corrections common in long-tail surveillance. Furthermore, to stabilize the optimization trajectory during extreme climatic anomalies, we enforced a strict gradient clipping protocol with a maximum norm of 5.0, effectively preventing gradient explosion across extended temporal backpropagation.

**Evaluation Protocol.** We rigorously quantified the forecasting performance using two primary metrics: Root Mean Squared Error (RMSE) and Pearson Correlation Coefficient (PCC). In the context of public health logistics, RMSE serves as a critical indicator for medical resource allocation (e.g., ICU beds) by penalizing large absolute deviations. Conversely, PCC evaluates the model’s capacity for phase alignment, which is essential for preemptive intervention during non-linear epidemic surges. Furthermore, recent environmental and climate-related forecasting studies have emphasized the value of decision-relevant evaluation beyond aggregate pointwise errors [29,30]. Following this broader evaluation perspective, we additionally evaluate Peak Timing Error (PTE) and Peak Magnitude Error (PME). PTE quantifies the absolute temporal phase shift (in weeks) between the predicted and actual epidemic peaks, while PME measures the relative magnitude deviation at the peak point. For each forecasting horizon, PTE and PME are computed over the complete held-out test trajectory for each node after assembling the horizon-specific predictions across the test period. Therefore, PTE measures the temporal displacement between the predicted and observed dominant peaks at the trajectory level, rather than the lead time of an individual forecasting sample. As a result, PTE can exceed the nominal forecasting horizon when the predicted trajectory places the dominant seasonal peak in a different part of the held-out test period.

All benchmarks were evaluated under a direct *h*-week-ahead forecasting protocol, with h∈{2,3,5,10,15}, to assess forecasting stability across progressively later target weeks under the standardized benchmark protocol. To ensure fair benchmarking and evaluation of variability across random initializations, we re-evaluated all reproducible baseline models under the same chronological train/validation/test split and five independent random seeds. Accordingly, all results in Table 3 and Table 4 are reported as mean ± standard deviation. All reproducible baselines were evaluated under the unified input window, target horizons, chronological split, preprocessing pipeline, and five-seed protocol. For uncertainty estimation, 90% prediction intervals were calibrated separately for each dataset–horizon setting using the empirical distribution of absolute validation residuals.

**Statistical Comparison.** CASRL and RAM-GNN were compared under the same fixed random seed using paired held-out forecast trajectories. At each forecast origin, squared errors were averaged across spatial nodes and evaluated using a two-sided Diebold–Mariano test with Bartlett-kernel HAC variance estimation, lag h−1, and the Harvey–Leybourne–Newbold small-sample correction. The resulting ten *p*-values across the two cohorts and five forecasting horizons were adjusted using the Holm procedure.

### 4.3. Comparative Performance Analysis

To evaluate the predictive stability of the proposed framework, we benchmark CASRL against representative spatio-temporal models across continuous horizons ranging from 2 to 15 weeks. Table 3 and Table 4 summarize the quantitative results on the US-States and US-Regions datasets.

The multi-seed comparisons show a horizon-dependent performance pattern. CASRL remains competitive at shorter forecast horizons and shows more evident advantages at later forecast horizons. On the US-States cohort, CASRL achieves the best 15-week RMSE and the strongest PCC at the 5-, 10-, and 15-week horizons. On the US-Regions cohort, CASRL obtains the best RMSE at the 5-, 10-, and 15-week horizons and achieves the strongest PCC at the 5- and 15-week horizons. These results indicate that the proposed climate-associated dual-topology design is particularly beneficial when near-term autoregressive signals become less sufficient.

The paired CASRL–RAM-GNN tests did not reject equal predictive accuracy after Holm correction across the evaluated dataset–horizon settings; complete test statistics are provided in Appendix B.

**Impact of Passive Climate Feature Concatenation.** To further separate the effect of model design from the mere availability of meteorological variables, we evaluated a RAM-GNN+Climate variant that directly concatenates the four climate covariates into the input space of RAM-GNN. On the US-States cohort at the 15-week horizon, this variant obtained an RMSE of 282.0 and a PCC of 0.795, underperforming both the original RAM-GNN and CASRL. This result suggests that directly appending meteorological covariates is not sufficient, and that the structured graph-view separation and adaptive fusion in CAGMP are important for using climate information effectively.

**Figure 3:**
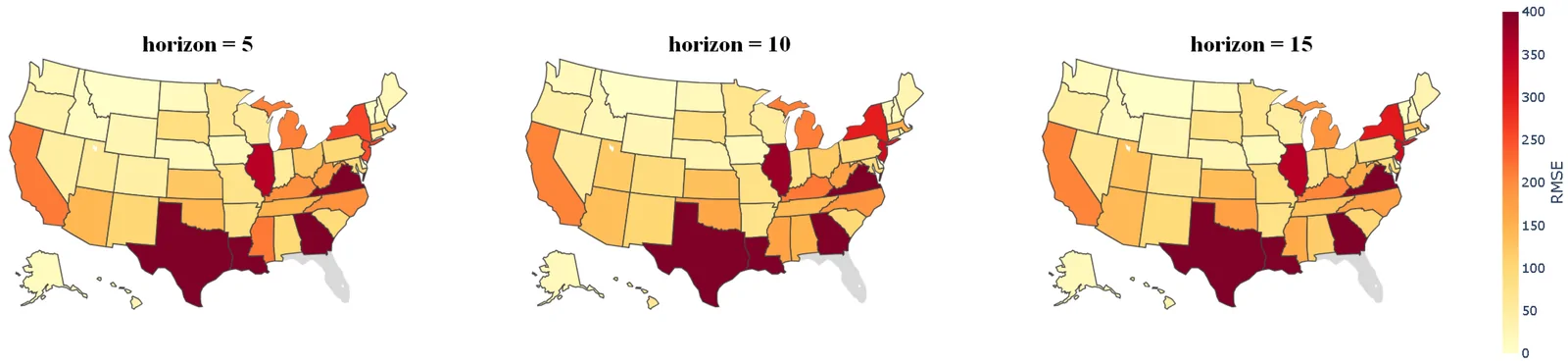
Spatial distribution of forecasting errors across US States. Choropleth maps visualize the geographic footprint of RMSE at 5-, 10-, and 15-week horizons to assess localized spatial divergence. Shorter horizons (e.g., 2 and 3 weeks) exhibit comparatively lower errors with less pronounced spatial differentiation and are thus omitted to emphasize later-horizon spatial error patterns.

**Spatial Robustness Analysis.** To visually investigate the spatial error propagation during forecasting at later forecast horizons, we first map the geographic distribution of prediction errors across the high-resolution state-level cohort (Figure 3). In STGNNs that primarily rely on historical incidence and fixed spatial assumptions, forecasting at later horizons may lead to accumulated spatial errors, particularly in geographically heterogeneous nodes. However, the generated state-level heatmaps reveal that the spatial error profile of CASRL does not show obvious localized error explosion up to the 15-week-ahead setting. Compared with rigid graph architectures, the forecasting errors remain comparatively stable across the US map.

**Figure 4:**
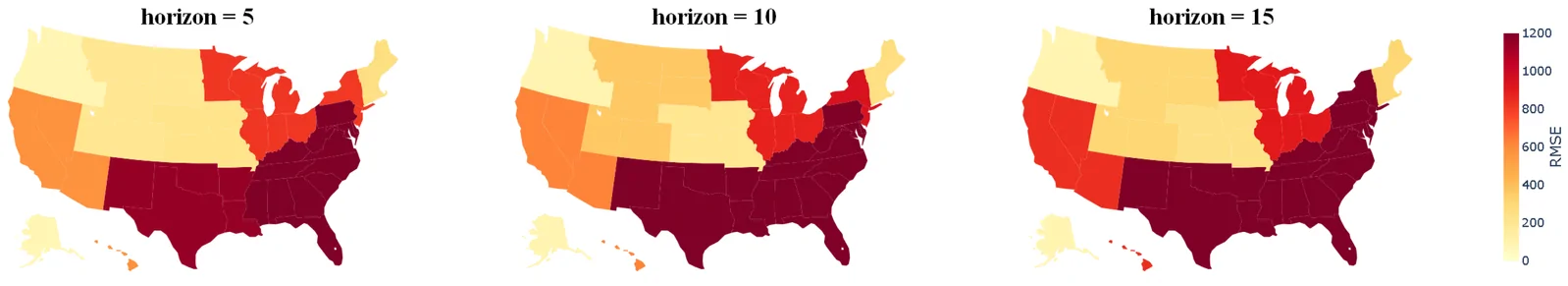
Spatial distribution of forecasting errors across HHS Regions. Choropleth maps visualize the regional RMSE footprint, evaluating macro-level spatial stability at later forecast horizons. Shorter horizons (e.g., 2 and 3 weeks) exhibit universally low errors and are thus omitted to emphasize later-horizon spatial error patterns.

This geographic stability is further corroborated at the macro-level in the US-Regions cohort (Figure 4). Despite the aggregation of distinct local transmission dynamics into broader administrative zones, the regional error footprint does not show obvious localized error explosion. This cross-scale pattern suggests that the dual-view CAGMP module provides a more stable spatial predictive representation across later forecast horizons.

**Figure 5:**
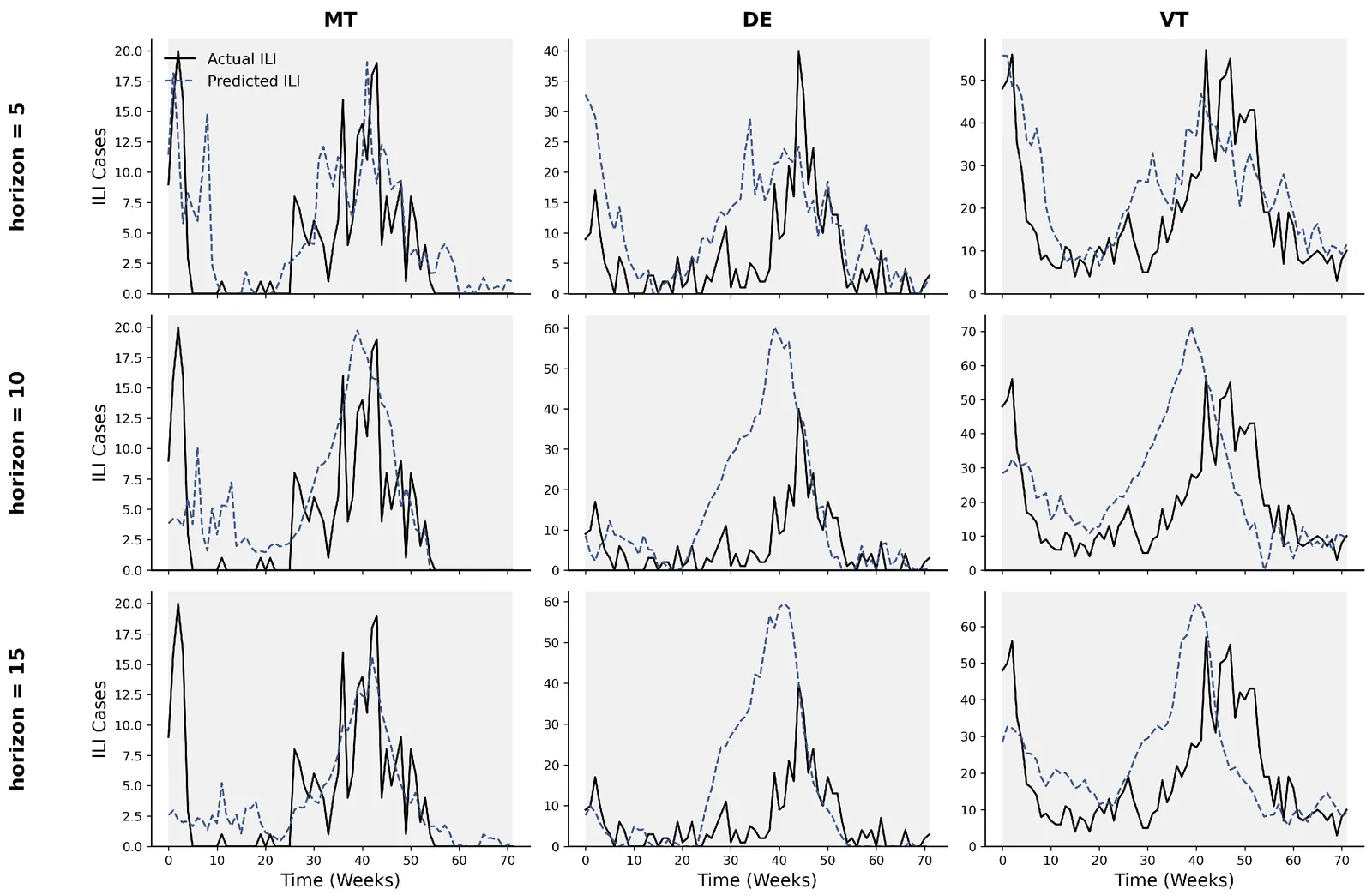
**Longitudinal trajectory forecasting across randomly selected US States.** The 3 × 3 grid displays complete held-out test-period trajectories for randomly selected US-State nodes (MT, DE, VT) across 5-, 10-, and 15-week forecasting horizons. Predicted ILI curves are shown in dashed blue, and ground-truth observations are shown in solid black.

**Figure 6:**
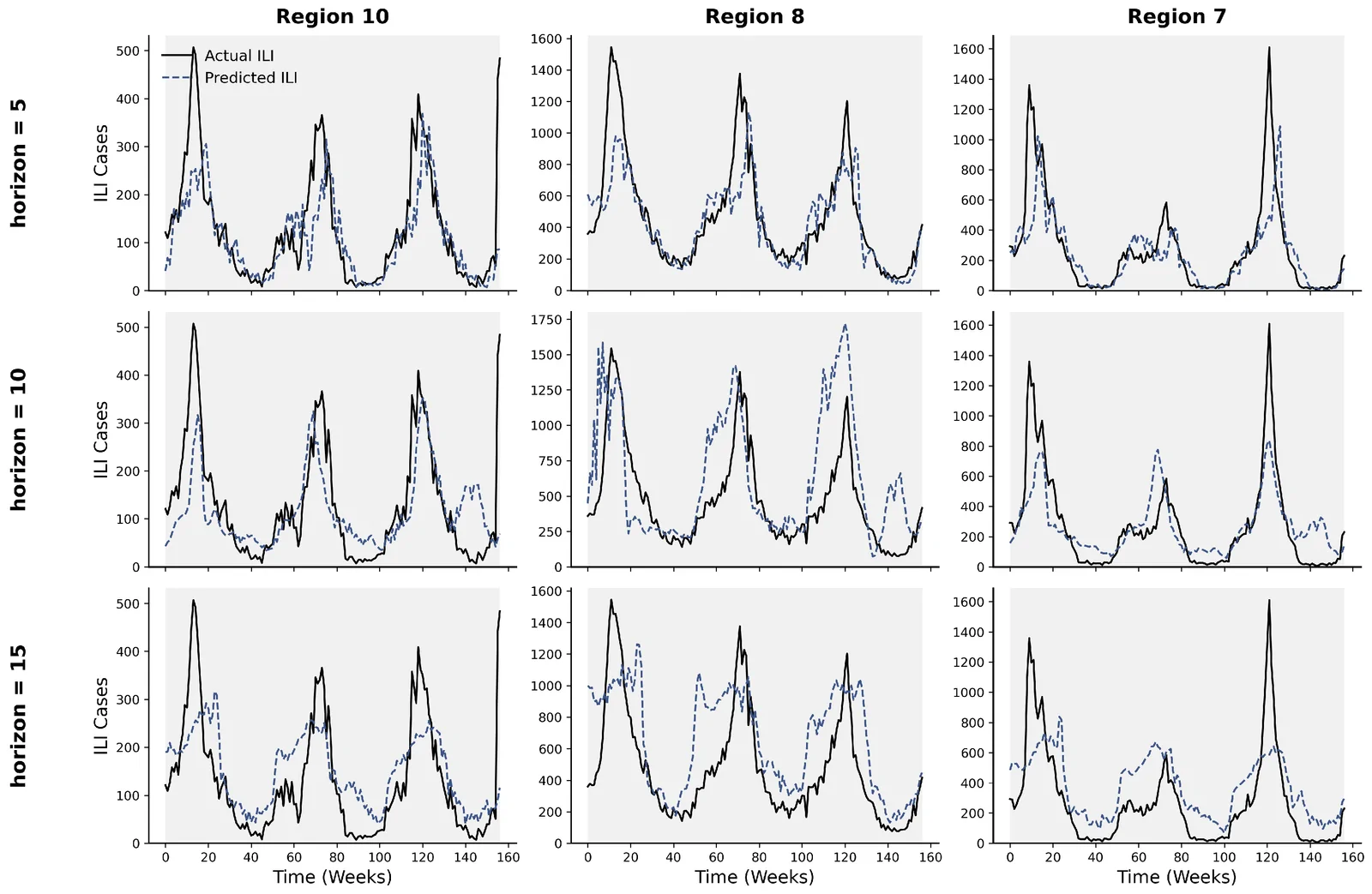
**Longitudinal trajectory forecasting across randomly selected HHS Regions.** The 3 × 3 grid displays complete held-out test-period trajectories for randomly selected HHS Regions (Regions 10, 8, and 7) across 5-, 10-, and 15-week forecasting horizons. Predicted ILI curves are shown in dashed blue, and ground-truth observations are shown in solid black.

**Temporal Trajectory and Phase Alignment.** Beyond aggregate volume estimation, anticipating the timing and phase of epidemic surges is important for operational preparedness. The state-level and region-level trajectories visualized in Figure 5 and Figure 6 are qualitative examples from randomly selected nodes over the complete held-out test period. These examples are used for trajectory-level visualization, while all-node spatial behavior is summarized by the spatial error maps in Figure 3 and Figure 4 across the 5-, 10-, and 15-week horizons. Complete node-wise trajectories for all HHS regions across the 2-, 3-, 5-, 10-, and 15-week forecasting horizons are provided in Supplementary Figures S1–S5, while the corresponding trajectories for all 49 US-State nodes are provided in Supplementary Figures S6–S10.

At shorter horizons (e.g., 5 weeks), CASRL demonstrates a tight coupling of both magnitude and phase, accurately tracing the sharp epidemic peaks. A recognized phenomenon in absolute-error-optimized temporal models is that extending the forecast horizon inherently induces a magnitude smoothing effect. As observed in the bottom rows (15-week horizon), the predicted absolute peak amplitudes experience expected attenuation. However, despite this amplitude smoothing, the predicted trajectories exhibit clear phase alignment with the ground-truth curves. The model generally preserves transition points, onset slopes, and peak timings without showing pronounced temporal lag in these representative trajectories. This cross-horizon temporal behavior suggests that SREE helps preserve informative historical epidemic states, supporting temporal phase stability even when forecasting up to 15 weeks in advance.

Beyond visual trajectory synchronization, we further quantified phase-related forecasting behavior using Peak Timing Error (PTE) and Peak Magnitude Error (PME) against RAM-GNN, a strong retrospective baseline, across the predefined forecasting horizons. As summarized in Table 5, CASRL shows competitive or improved peak-related performance in several later-horizon settings, particularly on the US-Regions cohort.

On the US-Regions dataset, CASRL reduces the 15-week PTE compared with RAM-GNN (67.8 vs. 75.1 weeks) and also yields a lower PME (0.2838 vs. 0.3675). On the US-States cohort, CASRL achieves lower PTE and PME at the 15-week horizon, although RAM-GNN remains competitive at shorter horizons. These results suggest that the proposed retrospective temporal encoding and climate-associated dual-topology message passing can improve phase-related forecasting behavior in selected later-horizon settings.

**Prediction Intervals.** The calibrated intervals achieve empirical coverage of 89.2–91.6% on US-States and 84.5–87.8% on US-Regions. Interval width increases with forecast horizon, particularly on the regional cohort (Table 6).

### 4.4. Ablation Study

To systematically evaluate the structural contributions of the proposed CASRL framework and isolate the components that improve predictive stability at later forecast horizons, we conduct comprehensive ablation studies on both the spatio-temporal encoding modules and the adaptive gating mechanisms.

**Effectiveness of Dual-Topology and Retrospective Encoding.** To evaluate the respective contributions of retrospective temporal encoding and dual-topology spatial modeling, we define four structural variants of the full model (Table 7). w/o SREE (Standard RNN) replaces the Self-Retrospective Epidemic Encoder (SREE) with a standard recurrent neural network while retaining both graph views. w/o $A_{\mathrm{cli}}$ (Incidence Graph Only) and w/o $A_{\mathrm{epi}}$ (Climate-Associated Graph Only) retain SREE but remove the climate-associated and incidence-associated graph views, respectively. The Single Mixed Graph variant concatenates the epidemic and meteorological representations before graph construction and learns one shared predictive topology, thereby removing the explicit separation and adaptive fusion of the two graph views.

Removing SREE increases the 15-week RMSE to 1051 on the US-Regions cohort, indicating reduced later-horizon performance without retrospective retrieval of informative historical states. Removing either predictive graph view also degrades performance in selected later-horizon settings: the *w/o* $A_{\mathrm{cli}}$ variant yields an RMSE of 1170 at 10 weeks on US-Regions, while the *w/o* $A_{\mathrm{epi}}$ variant reduces the 15-week PCC to 0.501. The Single Mixed Graph variant likewise performs less favorably than the complete dual-topology architecture at 15 weeks, with RMSE increasing from 212 to 258 on US-States and from 863 to 1000 on US-Regions. These results support the complementary contributions of retrospective temporal encoding and explicitly separated predictive graph views in CASRL.

Inspection of the retrospective weights shows that SREE distributes attention across the historical window rather than concentrating on a single lag. At h=15, the highest-weight lags are 15, 18, and 17 on US-States and 18, 16, and 14 on US-Regions, with normalized entropy values of 0.9992 and 0.9978, respectively.

**Effect of Climate Encoder Architecture.** RNN, GRU, and lightweight Transformer climate encoders were compared with all remaining CASRL components unchanged. GRU and Transformer are competitive at shorter horizons, whereas RNN achieves the best RMSE and PCC at both h=10 and h=15 on the two cohorts (Table 8). The RNN is therefore used as the climate encoder.

**Contribution of Meteorological Covariates.** To examine whether the retained meteorological variables provide useful predictive information, we conducted climate-covariate ablation experiments on both cohorts (Table 9). The “w/o all climate” variant removes all meteorological covariates, while the partial variants either retain temperature-related covariates only (TMAX + TMIN), retain precipitation- and wind-related covariates only (PRCP + AWND), or remove one covariate at a time.

The contribution of individual meteorological covariates varies across cohorts and forecast horizons. At h=15, PRCP has the largest effect on US-Regions: removing PRCP increases RMSE from 863 to 1137 and reduces PCC from 0.831 to 0.747. On US-States, removing AWND produces the largest PCC reduction, while AWND and TMIN produce the largest RMSE increases.

The “w/o all climate” configuration further confirms that an incidence-only variant of the framework can be trained and evaluated without meteorological inputs. However, removing the meteorological pathway reduces 15-week performance on both cohorts, with the largest degradation observed on the US-Regions cohort.

**Impact of Adaptive Temperature Scaling in Gated Fusion.** Furthermore, to evaluate the core innovation of the node-specific adaptive gating mechanism within the CAGMP module, we ablate the framework by replacing the learnable temperature scaling parameter (τ) with fixed scalar values (Fixed τ=0.3, τ=0.5, τ=0.7). As shown in Table 10, enforcing a fixed temperature constrains the model’s capacity to adjust the sharpness of its decision boundary. A static temperature applies a uniform gating variance across all nodes, limiting the model’s ability to adapt graph-view fusion across heterogeneous regions. Consequently, the fixed variants exhibit notable performance fluctuations across different horizons. In contrast, while fixed-temperature variants can occasionally offer a rigidly stable filtering mechanism that benefits short-term (2–3 weeks) predictions, the complete CASRL architecture with an adaptive τ shows its clearest benefit in predictive stability at later forecast horizons, particularly on the US-Regions dataset and in PCC-based phase alignment, although fixed-temperature variants may still obtain marginally lower RMSE in several short-horizon or dataset-specific settings. By learning the temperature parameter, the adaptive gate adjusts the relative contribution of incidence-associated and meteorological-view messages for each region.

Across HHS regions, the climate-view contribution is broadly distributed rather than concentrated in a small subset. At h=15, Regions 8 and 7 have the highest mean climate-view weight (0.736), while the values across all ten regions range only from 0.724 to 0.736. The climate-view weight also does not exhibit a consistent monotonic increase with forecast horizon. Its variation differs across CAGMP layers and cohorts, showing horizon-dependent graph-view weighting.

### 4.5. Sensitivity and Robustness Analysis

To examine both structural sensitivity and input robustness, we evaluated the effects of the historical look-back window (*W*), the number of CAGMP layers (*L*), and incomplete meteorological observations. These diagnostic analyses were conducted under one fixed validation-selected random seed to isolate the effects of controlled changes in model configuration or input completeness.

First, varying the look-back window (W∈{10,15,20,25,30}) reveals a distinct structural optimality, particularly at later forecast horizons (Table 11). Performance degrades when *W* is too constrained (e.g., US-Regions 15-week RMSE of 933 at W=10), as a truncated sequence fails to capture sufficient historical transmission momentum. Conversely, excessively long windows (e.g., US-Regions RMSE of 1190 at W=25) introduce distant, predictively irrelevant epidemiological noise, deteriorating the temporal representations. Overall, W=20 provides a favorable balance between preserving retrospective momentum and avoiding excessive historical noise in this setting.

Second, evaluating the layer depth (L∈{1,2,3,4,5}) confirms the necessity of controlled spatial message passing (Table 12). A shallow architecture (L=1, US-Regions 15-week RMSE 1142) lacks the required spatial receptive field to capture cross-regional meteorological-view predictive associations. However, stacking deeper layers (L≥3) triggers severe over-smoothing—a recognized phenomenon in graph neural networks—which heavily dilutes localized incidence signals and causes performance to plummet (e.g., US-States 15-week RMSE 304 at L=5). Thus, L=2 provides the effective spatial receptive field for aggregating cross-regional predictive information without compromising localized feature distinctiveness.

**Robustness to Missing Meteorological Observations.** We further evaluated CASRL under 10%, 30%, and 50% missing meteorological observations using five independently generated masks. Missingness was randomly introduced at the node-week level in the held-out test inputs, with all four meteorological variables jointly masked, followed by the same causal imputation and training-period normalization pipeline using a fixed trained checkpoint. The most pronounced sensitivity occurs in the later-horizon US-Regions setting: at h = 15, 50% missingness increases RMSE from 863 to 1206 and decreases PCC from 0.831 to 0.692. On US-States, the corresponding degradation is more moderate, from 212 to 242 in RMSE and from 0.912 to 0.882 in PCC.

### 4.6. Computational Efficiency Analysis

CASRL introduces additional computation for the climate encoder and dual-view graph modeling, while retaining millisecond-level online inference on both cohorts. Its peak GPU memory is comparable to RAM-GNN under the same profiling setting (Table 13).

## 5. Discussion

Influenza activity reflects the interplay of viral, host, behavioral, and environmental factors. Previous epidemiological studies have associated meteorological conditions, particularly temperature and absolute humidity, with influenza transmission intensity and seasonal timing. Consistent with this epidemiological context, our covariate ablations show that meteorological information provides complementary predictive value, although its contribution varies across cohorts and forecasting horizons.

Several epidemiological pathways may underlie these associations, including changes in viral persistence, host susceptibility, and seasonal contact environments. Within CASRL, the meteorological pathway translates observed weather conditions into a separate predictive graph view, allowing regional meteorological similarity to inform spatial forecasting without premature mixing with incidence representations. Together with the retrospective epidemic representation learned by SREE, this decoupled design provides complementary temporal and meteorological context that may contribute to the more stable performance observed in several later-horizon settings.

From a public-health perspective, later-horizon forecasts can provide additional lead time for surveillance prioritization, preparedness planning, and preliminary resource allocation.

When comparing multiple forecasting methods across several horizons, isolated numerical rankings should be interpreted cautiously because of the multiplicity of comparisons. Accordingly, the broad benchmark results are considered primarily in terms of recurring performance patterns across datasets and forecasting horizons rather than individual best-performing values. Where formal predictive-accuracy testing is conducted, multiplicity across the predefined dataset–horizon comparisons is controlled using the Holm procedure.

**Limitations.** The present framework relies on several assumptions regarding data representation and temporal generalizability. Weekly meteorological summaries derived from representative stations are assumed to provide informative proxies for regional environmental conditions, although this approximation may underrepresent within-region climatic heterogeneity. The chronological forecasting protocol further assumes that predictive relationships learned from the training period remain informative during the subsequent validation and test periods. In addition, the learned incidence-associated and meteorological-view graphs should be interpreted as data-driven predictive topologies rather than transmission, contact, or causal networks.

The temporal coverage of the current benchmarks introduces an additional limitation. Both datasets end in 2017 and therefore evaluate CASRL only under pre-COVID seasonal influenza conditions. Pandemic- and post-pandemic changes in surveillance practices, healthcare-seeking behavior, public-health interventions, and influenza seasonality may alter the underlying data distribution and require further evaluation using post-2020 surveillance records.

## 6. Conclusions

In this study, we presented CASRL, a Climate-Aware Self-Retrospective Representation Learning framework tailored for spatio-temporal epidemic forecasting across interconnected regions. To overcome the autoregressive information bottlenecks typical of standard sequence models, we proposed the Self-Retrospective Epidemic Encoder to preserve historically salient epidemic momentum within historical surveillance windows. Furthermore, departing from rigid, environmentally-blind spatial models, we introduced the Climate-Adaptive Graph Message Passing module. By utilizing high-resolution climate data as meteorological-view predictive context, CAGMP dynamically constructs a meteorological-view predictive topology. This joint architecture models delayed meteorological associations and adaptively integrates incidence-associated and meteorological-view graph representations for *h*-week-ahead forecasting.

Experiments on two public ILI benchmarks show that CASRL achieves competitive performance at shorter forecast horizons and clearer advantages at later forecast horizons, particularly for phase alignment and 15-week-ahead forecasting. These findings support the value of integrating retrospective epidemic representations with meteorological-view predictive context for improving spatio-temporal influenza forecasting.

## Figures and Tables

**Table 1:** Statistics of datasets: nodes, week, min, max, mean.

Dataset	Nodes	Weeks	Min	Max	Mean
US-States	49	360	0	9716	223
US-Regions	10	785	0	16,526	1009

**Table 2:** Serial and spatio-temporal dependence of weekly ILI.

Diagnostic	US-States	US-Regions
Lag-1 autocorrelation	0.921	0.945
Ljung–Box significant nodes	100%	100%
Mean weekly Moran’s *I*	0.045	−0.061
Spatio-temporal Moran’s *I*	0.718	0.808
Permutation *p*-value	0.001	0.001

**Table 3:** **Performance comparison under standardized benchmark horizons on the US-States cohort.** Forecasting accuracy is evaluated using RMSE and PCC across the predefined benchmark horizons of 2, 3, 5, 10, and 15 weeks. All reproducible baselines are re-evaluated under the same chronological split and five independent random seeds. Results are reported as mean ± standard deviation. Best results are highlighted in bold, and the second-best results are underlined.

	US-States				
RMSE (↓)	2	3	5	10	15
GAR [31]	162.0±0.2	199.3±0.6	246.8±0.2	341.1±0.4	368.5±0.3
AR [32]	171.7±1.1	212.2±0.8	264.1±0.7	332.2±0.5	354.4±0.6
VAR [33]	280.9±21.8	297.6±16.3	317.8±16.2	423.3±16.0	411.8±22.5
ARMA [34]	172.7±1.1	208.6±1.5	261.0±1.0	331.3±1.5	353.9±0.9
RNN [17]	159.2±0.9	193.8±3.6	225.8±1.2	306.9±8.8	351.1±6.3
LSTM [35]	161.2±1.2	197.9±0.7	226.0±2.6	310.9±2.1	350.2±6.8
RNN + Attn [36]	163.8±2.0	198.3±1.8	246.1±7.8	326.0±6.0	350.6±6.7
DCRNN [37]	174.9±7.7	208.9±7.7	261.5±7.1	320.1±4.2	325.9±3.5
CNNRNN-Res [38]	220.1±2.9	303.4±15.0	333.8±33.2	296.5±23.3	268.5±6.8
LSTNet [5]	192.9±5.0	232.4±6.8	283.1±12.0	310.6±11.8	319.4±12.8
ST-GCN [39]	209.4±5.6	227.3±2.3	260.5±4.6	296.0±2.2	301.3±5.4
Cola-GNN [7]	148.2±8.7	178.6±4.6	210.9±19.4	250.4±12.8	238.3±7.9
GAST [40]	142.3±4.3	179.8±4.1	236.0±12.7	257.2±12.2	248.5±7.8
RAM-GNN [20]	143.8±6.3	176.0±5.3	215.2±8.7	245.8±4.4	239.0±8.7
**CASRL (OURS)**	147.8±6.3	178.0±6.3	224.4±10.3	250.8±12.7	230.4±17.6
**PCC (↑)**	**2**	**3**	**5**	**10**	**15**
GAR [31]	0.938±0.000	0.906±0.000	0.861±0.000	0.725±0.000	0.696±0.000
AR [32]	0.933±0.000	0.902±0.001	0.850±0.000	0.738±0.001	0.687±0.002
VAR [33]	0.808±0.028	0.785±0.021	0.751±0.028	0.625±0.013	0.564±0.047
ARMA [34]	0.932±0.001	0.901±0.001	0.850±0.001	0.738±0.002	0.685±0.003
RNN [17]	0.944±0.000	0.917±0.003	0.890±0.001	0.789±0.013	0.722±0.017
LSTM [35]	0.944±0.000	0.915±0.000	0.890±0.001	0.780±0.005	0.728±0.013
RNN + Attn [36]	0.943±0.001	0.915±0.001	0.869±0.007	0.740±0.004	0.695±0.016
DCRNN [37]	0.938±0.007	0.912±0.009	0.868±0.011	0.808±0.011	0.816±0.009
CNNRNN-Res [38]	0.890±0.003	0.792±0.015	0.715±0.072	0.789±0.027	0.833±0.004
LSTNet [5]	0.925±0.003	0.882±0.006	0.807±0.018	0.759±0.024	0.800±0.016
ST-GCN [39]	0.898±0.006	0.881±0.002	0.835±0.005	0.798±0.011	0.799±0.009
Cola-GNN [7]	0.954±0.002	0.935±0.004	0.900±0.012	0.871±0.024	0.897±0.008
GAST [40]	0.954±0.002	0.927±0.004	0.859±0.023	0.837±0.022	0.854±0.016
RAM-GNN [20]	0.955±0.003	0.934±0.008	0.910±0.013	0.885±0.008	0.896±0.009
**CASRL (Ours)**	0.955±0.002	0.935±0.002	0.911±0.005	0.892±0.007	0.905±0.007

**Table 4:** **Performance comparison under standardized benchmark horizons on the US-Regions cohort.** Forecasting accuracy is evaluated using RMSE and PCC across the predefined benchmark horizons of 2, 3, 5, 10, and 15 weeks. All reproducible baselines are re-evaluated under the same chronological split and five independent random seeds. Results are reported as mean ± standard deviation. Best results are highlighted in bold, and the second-best results are underlined.

	US-Regions				
RMSE (↓)	2	3	5	10	15
GAR [31]	604.9±2.2	793.0±2.2	1060.7±5.1	1500.8±6.0	1624.5±18.9
AR [32]	624.0±3.0	811.4±2.2	1076.8±3.8	1447.9±2.5	1545.6±5.7
VAR [33]	705.6±21.0	863.9±16.3	1094.1±35.0	1337.9±15.1	1385.9±13.9
ARMA [34]	623.9±6.6	803.6±4.1	1061.0±7.5	1430.0±3.9	1539.6±3.3
RNN [17]	573.9±3.0	747.1±8.8	950.7±34.7	1433.9±31.6	1538.1±81.0
LSTM [35]	569.2±3.5	741.1±8.3	1036.5±16.9	1413.7±9.3	1553.0±122.5
RNN + Attn [36]	566.4±7.9	1050.8±383.0	1012.0±29.1	1439.6±92.8	1542.1±32.5
DCRNN [37]	774.1±22.1	935.0±14.2	1206.4±14.8	1520.6±6.1	1622.3±2.3
CNNRNN-Res [38]	595.5±7.8	781.0±35.8	990.5±45.6	1269.1±11.8	1333.9±25.9
LSTNet [5]	600.8±10.9	818.2±38.4	1071.4±65.3	1144.0±65.0	1252.1±179.9
ST-GCN [39]	706.9±13.6	826.2±13.9	1024.6±51.9	1113.4±55.3	1121.7±94.9
Cola-GNN [7]	529.2±7.5	673.6±13.2	867.0±43.6	997.4±111.6	1033.3±96.2
GAST [40]	490.5±7.9	649.5±19.7	923.6±40.2	1291.4±28.1	1359.8±47.7
RAM-GNN [20]	533.0±11.6	681.2±14.0	878.6±19.2	973.6±37.5	1000.4±27.5
**CASRL (OURS)**	536.0±22.8	673.5±19.7	847.3±46.0	973.0±92.2	931.5±32.1
**PCC (↑)**	**2**	**3**	**5**	**10**	**15**
GAR [31]	0.924±0.001	0.870±0.001	0.782±0.001	0.578±0.018	0.511±0.038
AR [32]	0.922±0.001	0.871±0.001	0.788±0.001	0.613±0.004	0.556±0.003
VAR [33]	0.893±0.007	0.839±0.010	0.732±0.020	0.561±0.012	0.514±0.015
ARMA [34]	0.920±0.002	0.871±0.001	0.786±0.002	0.612±0.002	0.552±0.005
RNN [17]	0.934±0.001	0.888±0.001	0.827±0.009	0.596±0.035	0.532±0.028
LSTM [35]	0.934±0.000	0.891±0.001	0.817±0.004	0.673±0.025	0.518±0.035
RNN + Attn [36]	0.935±0.001	0.743±0.188	0.813±0.006	0.592±0.034	0.561±0.033
DCRNN [37]	0.892±0.006	0.843±0.006	0.760±0.008	0.613±0.005	0.574±0.009
CNNRNN-Res [38]	0.925±0.002	0.870±0.006	0.784±0.019	0.607±0.011	0.569±0.014
LSTNet [5]	0.936±0.002	0.881±0.009	0.794±0.024	0.718±0.041	0.670±0.044
ST-GCN [39]	0.902±0.004	0.872±0.002	0.823±0.017	0.765±0.008	0.756±0.033
Cola-GNN [7]	0.942±0.002	0.907±0.004	0.855±0.016	0.800±0.036	0.791±0.030
GAST [40]	0.945±0.001	0.904±0.003	0.828±0.007	0.611±0.041	0.562±0.069
RAM-GNN [20]	0.942±0.002	0.905±0.003	0.851±0.010	0.807±0.024	0.796±0.005
**CASRL (Ours)**	0.942±0.005	0.907±0.003	0.862±0.006	0.808±0.035	0.822±0.015

**Table 5:** **Quantitative evaluation of decision-relevant phase alignment metrics.** Peak Timing Error (PTE, absolute weeks) and Peak Magnitude Error (PME, relative deviation) are reported across predefined forecasting horizons. Lower values indicate better peak timing or magnitude estimation. Best values are highlighted in bold.

	US-States					US-Regions				
PTE (↓)	2	3	5	10	15	2	3	5	10	15
RAM-GNN [20]	5.47	**4.37**	**4.41**	**4.12**	4.16	18.30	35.5	25.9	54.1	75.1
CASRL (Ours)	**5.37**	5.27	7.08	4.31	**3.76**	**13.00**	**24.6**	**25.8**	**44.4**	**67.8**
**PME (↓)**	**2**	**3**	**5**	**10**	**15**	**2**	**3**	**5**	**10**	**15**
RAM-GNN [20]	0.2578	0.3087	0.4581	**0.5312**	0.5461	0.0918	0.1557	0.4152	0.5295	0.3675
CASRL (Ours)	**0.2574**	**0.3005**	**0.4463**	0.5650	**0.4987**	**0.0571**	**0.1132**	**0.3430**	**0.5120**	**0.2838**

**Table 6:** Empirically calibrated prediction intervals with a nominal 90% coverage level.

Dataset	Metric	2	3	5	10	15
US-States	Coverage (%)	91.6	91.5	91.0	89.2	89.3
	Norm. width	0.029	0.035	0.044	0.043	0.042
US-Regions	Coverage (%)	84.5	85.3	86.4	87.8	85.9
	Norm. width	0.065	0.082	0.113	0.158	0.153

**Table 7:** Ablation study on the temporal encoding and spatial message passing modules under one fixed validation-selected random seed. To isolate the temporal contribution, the “w/o SREE” variant replaces the retrospective encoder with a standard RNN while keeping both spatial graphs intact. Conversely, the “w/o $A_{cli}$” and “w/o $A_{epi}$” variants retain the SREE module but isolate the impact of the dual-topology spatial message passing. The Single Mixed Graph variant concatenates the epidemic and meteorological representations before constructing one shared predictive topology. Best results are highlighted in bold.

	US-States					US-Regions				
RMSE (↓)	2	3	5	10	15	2	3	5	10	15
w/o SREE	142	**162**	222	251	**206**	547	715	870	1109	1051
w/o $A_{cli}$	178	177	**215**	237	237	575	692	843	1170	1098
w/o $A_{epi}$	**141**	174	222	254	240	**546**	741	967	1210	1446
Single Mixed Graph	162	193	244	244	258	564	704	871	1071	1000
CASRL	149	179	231	**233**	212	560	**663**	**861**	**874**	**863**
**PCC (↑)**	**2**	**3**	**5**	**10**	**15**	**2**	**3**	**5**	**10**	**15**
w/o SREE	**0.956**	**0.939**	0.896	0.889	0.897	0.939	0.896	0.844	0.766	0.823
w/o $A_{cli}$	0.925	0.935	0.905	0.883	0.861	0.934	0.902	0.855	0.758	0.805
w/o $A_{epi}$	0.955	0.934	0.902	0.877	0.892	**0.940**	0.881	0.798	0.762	0.501
Single Mixed Graph	0.947	0.923	0.900	0.871	0.875	0.937	0.899	0.852	0.766	0.810
CASRL	0.953	0.936	**0.906**	**0.899**	**0.912**	0.937	**0.908**	**0.861**	**0.837**	**0.831**

**Table 8:** **Comparison of alternative climate sequence encoders within CASRL.** The comparison is conducted under one fixed validation-selected random seed. Only the Climate Feature Encoder is replaced. Best results are highlighted in bold.

	US-Regions					US-States				
RMSE (↓)	2	3	5	10	15	2	3	5	10	15
GRU	**557**	681	**847**	941	889	150	**170**	227	252	242
Transformer	561	701	917	914	1002	153	176	**210**	240	254
RNN (default)	560	**663**	861	**874**	**863**	**149**	179	231	**233**	**212**
**PCC (↑)**	**2**	**3**	**5**	**10**	**15**	**2**	**3**	**5**	**10**	**15**
GRU	**0.938**	0.903	0.857	0.831	0.829	0.950	**0.936**	**0.911**	0.883	0.888
Transformer	0.935	0.901	0.848	0.829	0.781	0.950	0.935	0.907	0.893	0.891
RNN (default)	0.937	**0.908**	**0.861**	**0.837**	**0.831**	**0.953**	**0.936**	0.906	**0.899**	**0.912**

**Table 9:** Climate-covariate ablation under one fixed validation-selected random seed. The “w/o all climate” variant removes all meteorological covariates. The partial variants retain only selected covariate groups or remove one meteorological covariate at a time. Best results are highlighted in bold.

	US-States					US-Regions				
RMSE (↓)	2	3	5	10	15	2	3	5	10	15
w/o all climate	172	205	**226**	257	222	560	717	962	1122	1108
TMAX + TMIN only	150	**179**	239	236	225	**558**	693	862	1052	1014
PRCP + AWND only	171	208	238	257	235	563	669	863	954	914
w/o TMAX	163	212	241	238	226	569	722	864	919	977
w/o TMIN	166	209	245	233	230	572	687	869	956	921
w/o PRCP	150	185	234	239	223	559	675	**861**	899	1137
w/o AWND	164	217	227	238	230	564	716	**861**	970	968
Full CASRL	**149**	**179**	231	**233**	**212**	560	**663**	**861**	**874**	**863**
**PCC (↑)**	**2**	**3**	**5**	**10**	**15**	**2**	**3**	**5**	**10**	**15**
w/o all climate	0.938	0.910	0.896	0.876	0.898	0.937	0.896	0.851	0.789	0.791
TMAX + TMIN only	0.952	**0.937**	0.881	0.898	0.906	0.934	0.897	**0.863**	0.776	0.807
PRCP + AWND only	0.937	0.906	0.883	0.865	0.892	**0.938**	0.906	0.862	0.808	0.810
w/o TMAX	0.941	0.902	0.885	0.896	0.911	0.936	0.896	0.859	0.821	0.799
w/o TMIN	0.940	0.908	0.863	**0.899**	0.903	0.936	0.902	0.859	0.810	0.811
w/o PRCP	**0.953**	0.932	0.876	0.897	**0.914**	**0.938**	0.904	0.857	0.818	0.747
w/o AWND	0.939	0.905	**0.907**	0.895	0.885	0.934	0.895	0.859	0.783	0.787
Full CASRL	**0.953**	0.936	0.906	**0.899**	0.912	0.937	**0.908**	0.861	**0.837**	**0.831**

**Table 10:** Ablation study on the temperature scaling parameter within the CAGMP module under one fixed validation-selected random seed. Fixed scalar values (τ=0.3,0.5,0.7) are compared against the proposed learnable adaptive temperature. Best results are highlighted in bold.

	US-States					US-Regions				
RMSE (↓)	2	3	5	10	15	2	3	5	10	15
τ=0.3	161	192	236	235	210	**538**	682	862	1226	1087
τ=0.5	**141**	**160**	223	255	216	559	673	929	1096	1020
τ=0.7	142	162	**222**	321	**206**	555	720	870	1104	980
CASRL (Adaptive τ)	149	179	231	**233**	212	560	**663**	**861**	**874**	**863**
**PCC (↑)**	**2**	**3**	**5**	**10**	**15**	**2**	**3**	**5**	**10**	**15**
τ=0.3	0.941	0.922	0.864	0.873	0.903	**0.940**	0.907	0.845	0.763	0.769
τ=0.5	**0.956**	**0.940**	0.895	0.885	0.894	0.938	0.907	0.835	0.779	0.792
τ=0.7	**0.956**	0.939	0.896	0.798	0.897	0.938	0.902	0.844	0.763	0.810
CASRL (Adaptive τ)	0.953	0.936	**0.906**	**0.899**	**0.912**	0.937	**0.908**	**0.861**	**0.837**	**0.831**

**Table 11:** Sensitivity analysis on the historical look-back window size (*W*). To strictly isolate structural impacts from initialization variance, evaluations were conducted under a fixed validation-selected random seed. Best results are highlighted in bold.

	US-States					US-Regions				
RMSE (↓)	2	3	5	10	15	2	3	5	10	15
W=10	150	173	197	235	243	573	714	907	1084	933
W=15	**136**	174	190	256	241	566	695	907	1283	911
W=20 (CASRL)	149	179	231	**233**	**212**	560	**663**	861	**874**	**863**
W=25	150	183	222	248	262	**525**	669	**767**	983	1190
W=30	143	**172**	**187**	240	250	541	722	937	1030	1162
**PCC (↑)**	**2**	**3**	**5**	**10**	**15**	**2**	**3**	**5**	**10**	**15**
W=10	0.955	**0.938**	0.925	0.872	0.889	0.935	0.894	0.857	0.807	0.805
W=15	**0.959**	0.935	0.925	0.875	0.895	0.936	0.900	0.855	0.721	**0.840**
W=20 (CASRL)	0.953	0.936	0.906	**0.899**	**0.912**	0.937	**0.908**	0.861	**0.837**	0.831
W=25	0.951	0.935	0.921	0.885	0.901	**0.942**	0.907	**0.882**	0.776	0.836
W=30	0.953	0.937	**0.927**	0.892	0.907	0.941	0.895	0.848	0.842	0.799

**Table 12:** Sensitivity analysis on the number of CAGMP layers (*L*). To strictly isolate structural impacts from initialization variance, evaluations were conducted under a fixed validation-selected random seed. Best results are highlighted in bold.

	US-States					US-Regions				
RMSE (↓)	2	3	5	10	15	2	3	5	10	15
L=1	157	186	227	258	257	550	682	859	1114	1142
L=2 (CASRL)	149	**179**	231	**233**	**212**	560	**663**	861	**874**	**863**
L=3	**147**	181	233	279	238	566	738	952	1178	1045
L=4	148	185	210	256	256	**521**	721	**804**	1001	1057
L=5	160	195	**206**	277	304	565	711	814	1121	1163
**PCC (↑)**	**2**	**3**	**5**	**10**	**15**	**2**	**3**	**5**	**10**	**15**
L=1	0.951	0.934	0.904	0.860	0.887	0.940	**0.908**	0.865	0.750	0.731
L=2 (CASRL)	0.953	**0.936**	0.906	**0.899**	**0.912**	0.937	**0.908**	0.861	**0.837**	0.831
L=3	**0.955**	0.935	**0.917**	0.881	0.910	0.936	0.896	0.849	0.792	**0.841**
L=4	0.951	0.926	**0.917**	0.866	0.884	**0.945**	0.894	**0.891**	0.830	0.799
L=5	0.947	0.920	0.911	0.824	0.844	0.934	0.900	0.867	0.767	0.782

**Table 13:** Computational cost relative to RAM-GNN.

Model	Parameters	Fwd + Bwd	Online Inf.	Peak Memory
	States/Regions	ms/Batch	ms/Sample	MiB
RAM-GNN [20]	4546/2163	39.8/18.1	13.5/5.1	91/38
CASRL (Ours)	8263/3661	49.0/25.2	17.0/8.3	78/38

## Data Availability

The data used in this study are publicly available from the CDC FluView at https://gis.cdc.gov/grasp/fluview/fluportaldashboard.html (accessed on 16 August 2026) and the NOAA Global Historical Climatology Network Daily (GHCN-Daily) at https://www.ncei.noaa.gov/products/land-based-station/global-historical-climatology-network-daily (accessed on 16 August 2026).

## Supplementary Materials

The following supporting information can be downloaded at: https://www.mdpi.com/article/10.3390/tropicalmed11090240/s1, Figure S1: Complete region-level trajectory visualization of actual ILI and CASRL predictions at the 2-week forecasting horizon in the US-Regions cohort; Figure S2: Complete region-level trajectory visualization of actual ILI and CASRL predictions at the 3-week forecasting horizon in the US-Regions cohort; Figure S3: Complete region-level trajectory visualization of actual ILI and CASRL predictions at the 5-week forecasting horizon in the US-Regions cohort; Figure S4: Complete region-level trajectory visualization of actual ILI and CASRL predictions at the 10-week forecasting horizon in the US-Regions cohort; Figure S5: Complete region-level trajectory visualization of actual ILI and CASRL predictions at the 15-week forecasting horizon in the US-Regions cohort; Figure S6: Complete state-level trajectory visualization of actual ILI and CASRL predictions at the 2-week forecasting horizon in the US-States cohort; Figure S7: Complete state-level trajectory visualization of actual ILI and CASRL predictions at the 3-week forecasting horizon in the US-States cohort; Figure S8: Complete state-level trajectory visualization of actual ILI and CASRL predictions at the 5-week forecasting horizon in the US-States cohort; Figure S9: Complete state-level trajectory visualization of actual ILI and CASRL predictions at the 10-week forecasting horizon in the US-States cohort; Figure S10: Complete state-level trajectory visualization of actual ILI and CASRL predictions at the 15-week forecasting horizon in the US-States cohort.

## Appendix A. Chronological Split and Rolling-Window Protocol

To clarify the chronological evaluation protocol, we provide the rolling-window construction and split assignment used in all experiments. All datasets were divided chronologically into training, validation, and test periods following a 60%/20%/20% protocol. For the US-States cohort with 360 weeks, weeks 0–215, 216–287, and 288–359 were used for training, validation, and testing, respectively. For the US-Regions cohort with 785 weeks, weeks 0–470, 471–627, and 628–784 were used for training, validation, and testing, respectively.

For each forecasting horizon h∈{2,3,5,10,15}, we use a direct *h*-step-ahead rolling-window forecasting protocol. Each sample is constructed from a historical look-back window of W=20 weeks, where the model observes ILI and meteorological sequences from weeks t−W+1 to *t* and directly predicts the ILI values of all spatial nodes at the future target week t+h. The model does not recursively forecast each intermediate week; instead, each horizon defines a separate target-week prediction task.

The split assignment is determined by the target week t+h, rather than by the input window alone. Therefore, the number of training samples decreases as *h* increases because longer forecasting horizons require earlier forecast origins to keep the target week within the training period. In contrast, validation and test counts remain constant because their target-week blocks are fixed and have sufficient preceding observations to construct complete 20-week input windows.

**Table A1:** Rolling-window sample counts under the chronological 60%/20%/20% split. Window size is set to W=20, and each sample is assigned according to the target week t+h.

Dataset	*N*	*h*	Train	Val.	Test
US-States	360	2	195	72	72
US-States	360	3	194	72	72
US-States	360	5	192	72	72
US-States	360	10	187	72	72
US-States	360	15	182	72	72
US-Regions	785	2	450	157	157
US-Regions	785	3	449	157	157
US-Regions	785	5	447	157	157
US-Regions	785	10	442	157	157
US-Regions	785	15	437	157	157

We further report split-wise CASRL diagnostic performance on the training, validation, and held-out test periods. This diagnostic is intended to verify the chronological split, validation-based model selection, and held-out test evaluation. The official multi-seed benchmark comparisons remain those reported in the main result tables.

Because each forecasting horizon defines a different target-week prediction task and a different set of admissible forecast origins, the train/validation/test metrics in Table A2 are intended only as split-wise protocol diagnostics and should not be interpreted as repeated evaluations of the same prediction task.

**Table A2:** Split-wise CASRL diagnostic performance across chronological splits under one validation-selected run. RMSE and PCC are reported for each forecasting horizon to verify the training, validation, and held-out test evaluation protocol. Different horizons correspond to different target-week prediction tasks.

Dataset	*h*	Train		Validation		Test	
		RMSE	PCC	RMSE	PCC	RMSE	PCC
US-States	2	108.23	0.9554	201.30	0.9201	149.89	0.9529
US-States	3	127.72	0.9330	242.26	0.8837	179.35	0.9359
US-States	5	154.02	0.9013	285.70	0.8459	231.88	0.9057
US-States	10	162.02	0.8894	269.82	0.8593	233.31	0.8993
US-States	15	132.57	0.9309	259.63	0.8671	212.66	0.9123
US-Regions	2	521.78	0.9167	377.03	0.9568	560.07	0.9371
US-Regions	3	696.87	0.8456	484.89	0.9262	663.42	0.9076
US-Regions	5	805.09	0.7881	653.60	0.8649	861.23	0.8610
US-Regions	10	992.11	0.6581	811.01	0.7843	874.49	0.8372
US-Regions	15	777.36	0.8066	757.85	0.8107	863.57	0.8312

## Appendix B. Statistical Significance Analysis

The complete paired predictive-accuracy results between CASRL and RAM-GNN are reported in Table A3. Negative DM-HLN statistics indicate lower average squared forecast error for CASRL.

**Table A3:** Paired predictive-accuracy tests for CASRL and RAM-GNN.

	US-States					US-Regions				
Metric	2	3	5	10	15	2	3	5	10	15
DM-HLN	0.795	0.941	0.487	−1.169	−1.005	−0.925	−0.171	−1.816	0.148	−1.056
Raw *p*	0.4293	0.3502	0.6280	0.2464	0.3184	0.3564	0.8644	0.0714	0.8829	0.2925
Holm *p*	1.0000	1.0000	1.0000	1.0000	1.0000	1.0000	1.0000	0.7137	1.0000	1.0000
