# Supplementary material for: Climate-Aware Self-Retrospective Representation Learning for Spatio-Temporal Epidemic Forecasting
Source: Trop Med Infect Dis. 2026 Aug 24;11(9):240. doi: 10.3390/tropicalmed11090240 (PMC13611530; doi:10.3390/tropicalmed11090240)
Supplement: Supplementary file 1 [file tropicalmed-11-00240-s001.zip › tropicalmed-4453475-supplementary.pdf]

Supplementary Material

Title: Climate-Aware Self-Retrospective Representation Learning for Spatio-Temporal Epidemic Forecasting

Complete Node-Wise Trajectory Visualizations:

This supplementary document presents complete node-wise trajectories of observed ILI and CASRL predictions for all 49 US-State nodes and all 10 HHS Region nodes at the 2-, 3-, 5-, 10-, and 15-week forecasting horizons.

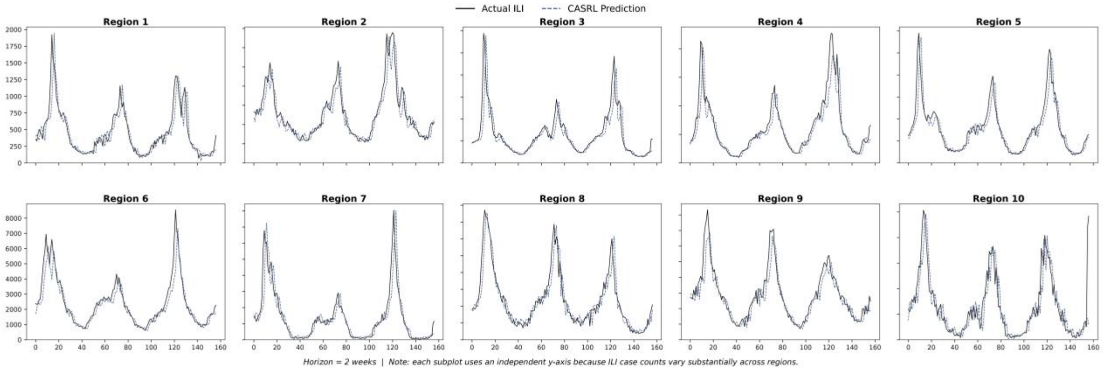

Figure S1: Complete region-level trajectory visualization of actual ILI and CASRL predictions at the 2-week forecasting horizon in the US-Regions cohort.

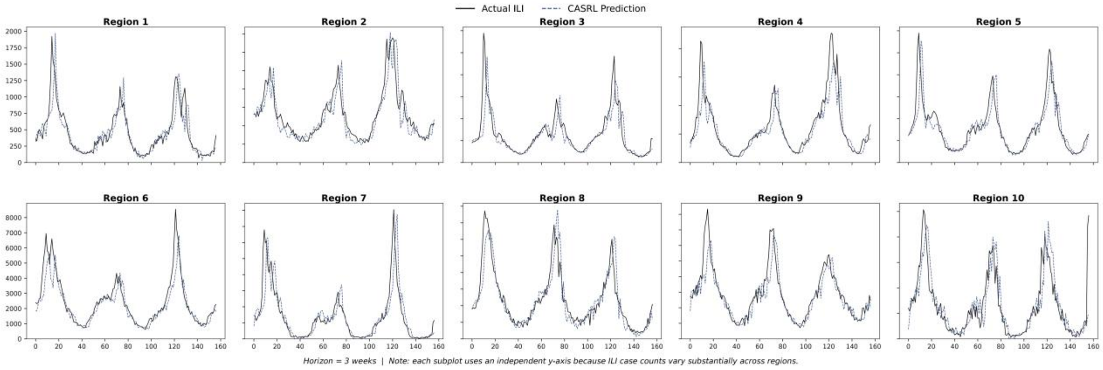

Figure S2: Complete region-level trajectory visualization of actual ILI and CASRL predictions at the 3-week forecasting horizon in the US-Regions cohort.

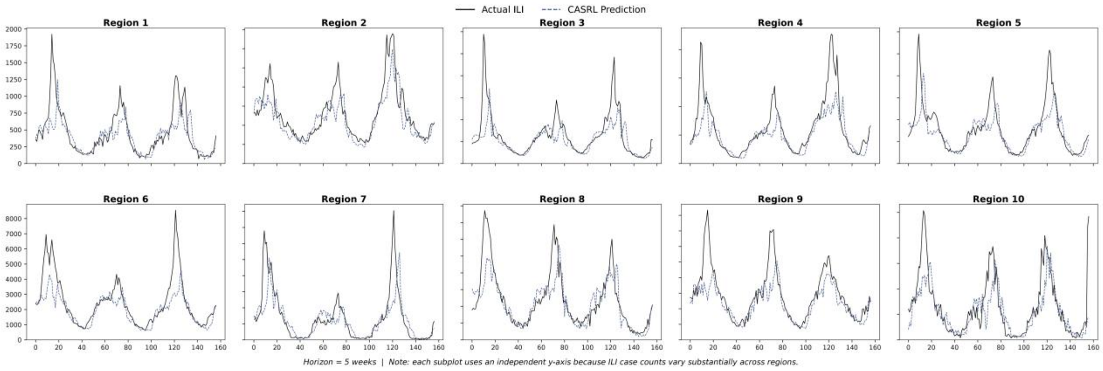

Figure S3: Complete region-level trajectory visualization of actual ILI and CASRL predictions at the 5-week forecasting horizon in the US-Regions cohort.

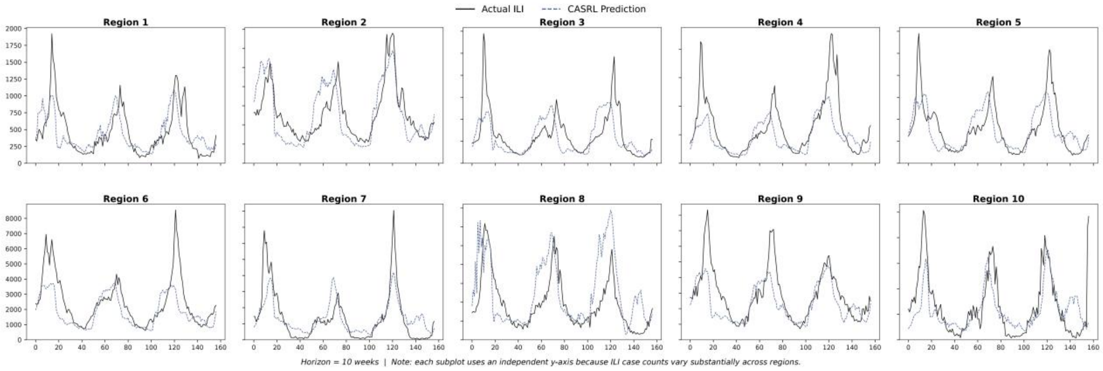

Figure S4: Complete region-level trajectory visualization of actual ILI and CASRL predictions at the 10-week forecasting horizon in the US-Regions cohort.

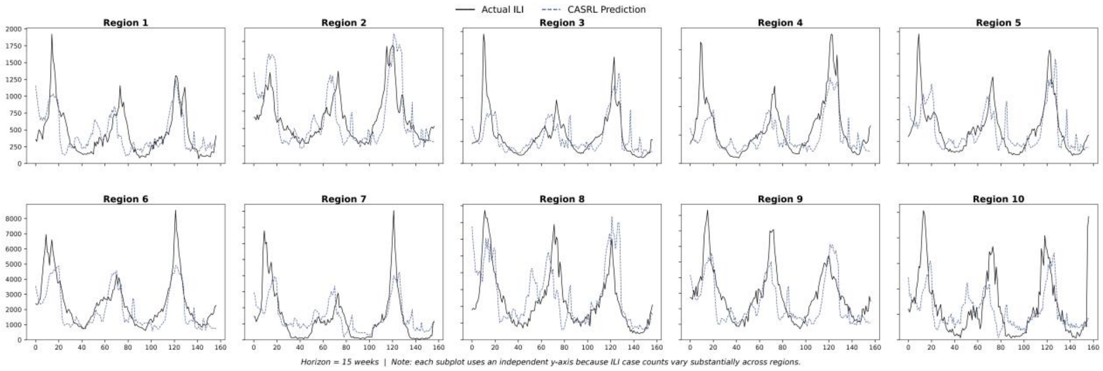

Figure S5: Complete region-level trajectory visualization of actual ILI and CASRL predictions at the 15-week forecasting horizon in the US-Regions cohort.

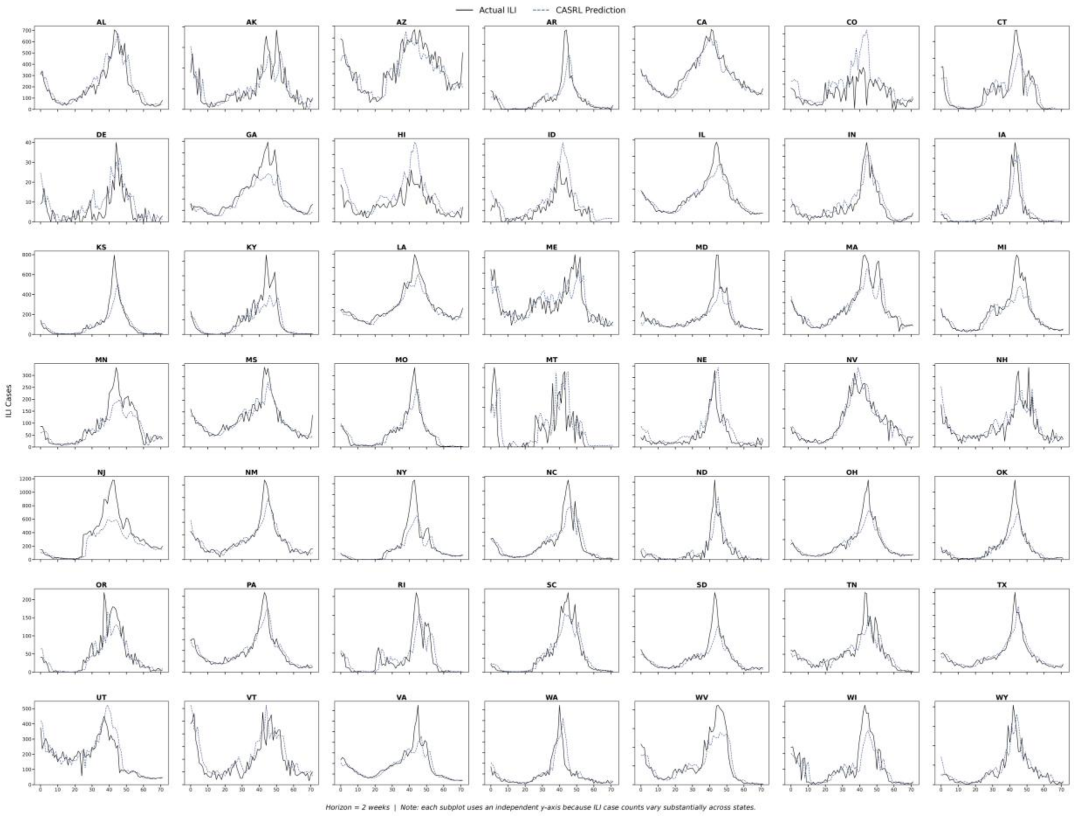

Figure S6: Complete state-level trajectory visualization of actual ILI and CASRL predictions at the 2-week forecasting horizon in the US-States cohort.

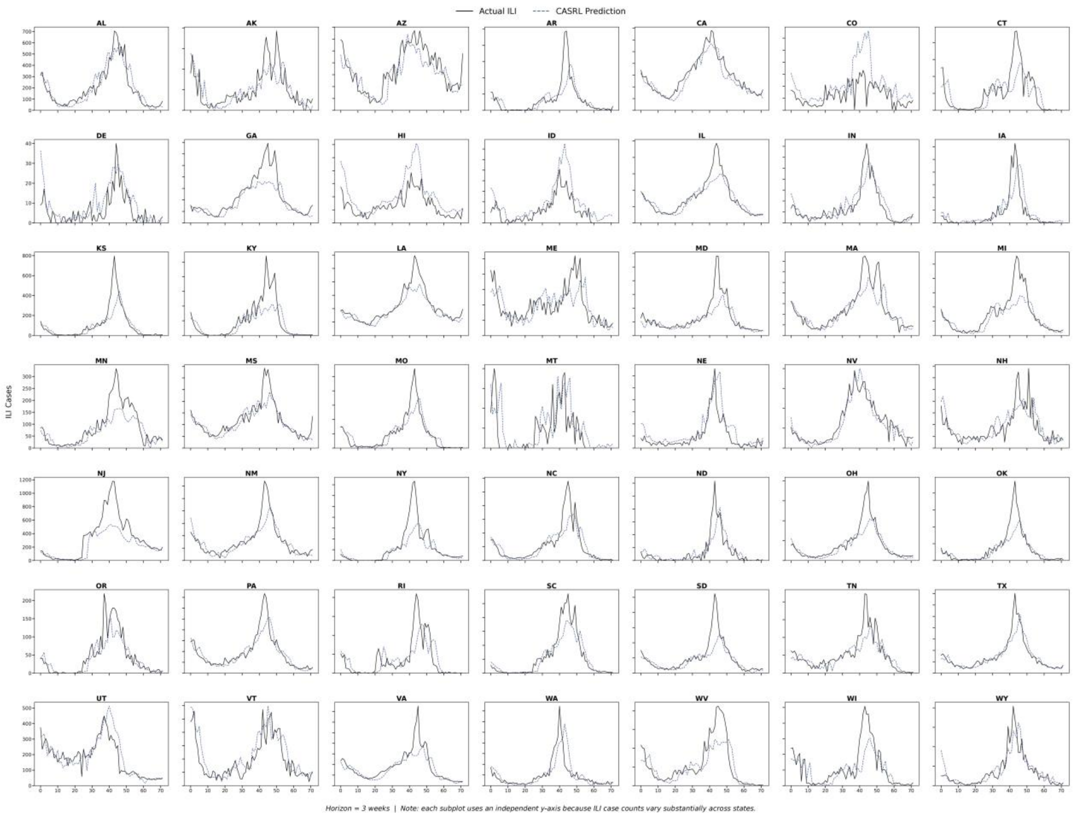

Figure S7: Complete state-level trajectory visualization of actual ILI and CASRL predictions at the 3-week forecasting horizon in the US-States cohort.

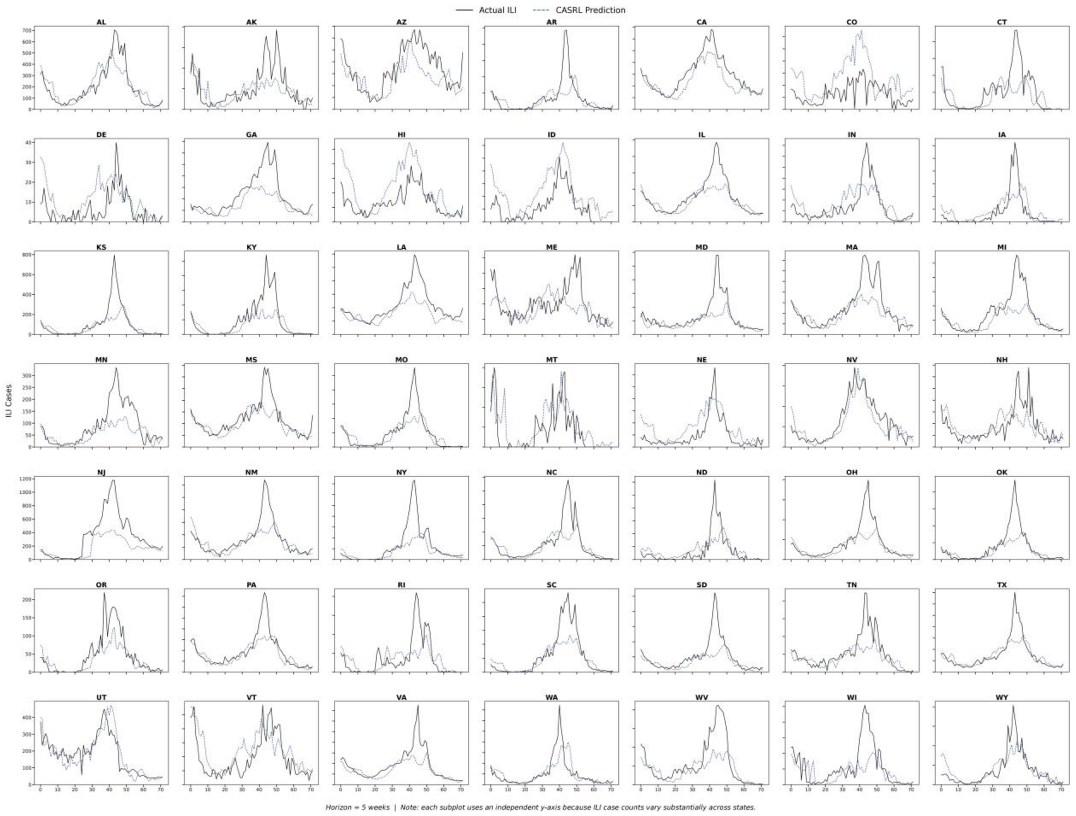

Figure S8: Complete state-level trajectory visualization of actual ILI and CASRL predictions at the 5-week forecasting horizon in the US-States cohort.

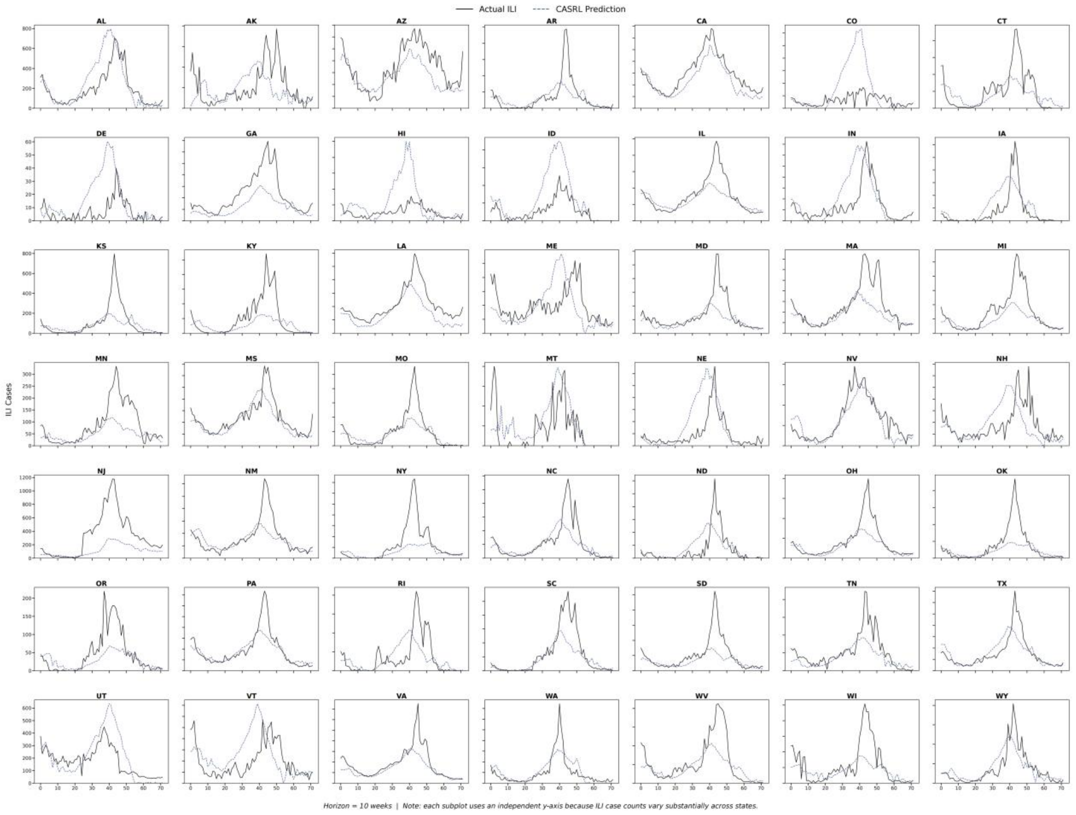

Figure S9: Complete state-level trajectory visualization of actual ILI and CASRL predictions at the 10-week forecasting horizon in the US-States cohort.

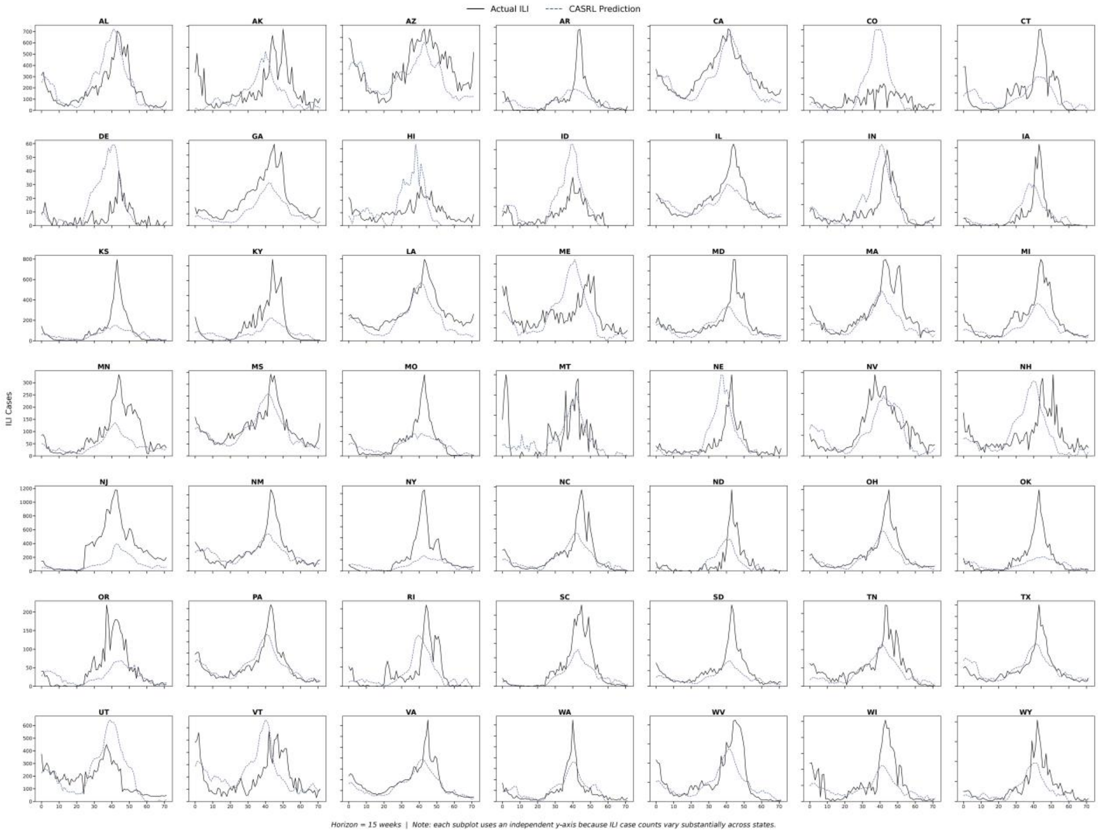

Figure S10: Complete state-level trajectory visualization of actual ILI and CASRL predictions at the 15-week forecasting horizon in the US-States cohort.
